# Overall photosynthesis of H_2_O_2_ by an inorganic semiconductor

**DOI:** 10.1038/s41467-022-28686-x

**Published:** 2022-02-24

**Authors:** Tian Liu, Zhenhua Pan, Junie Jhon M. Vequizo, Kosaku Kato, Binbin Wu, Akira Yamakata, Kenji Katayama, Baoliang Chen, Chiheng Chu, Kazunari Domen

**Affiliations:** 1 Faculty of Agriculture, Life, and Environmental Sciences, Zhejiang University, 310058 Hangzhou, China; 2 Department of Applied Chemistry, Faculty of Science and Technology, Chuo University, 1-13-27 Kasuga, Bunkyo, Tokyo 112-8551 Japan; 3 Research Initiative for Supra-Materials, Shinshu University, 4-17-1 Wakasato, Nagano-shi, Nagano, 380-8553 Japan; 4 Graduate School of Engineering, Toyota Technological Institute, 2-12-1, Hisakata, Tempaku, Nagoya, 468-8511 Japan; 5 Office of University Professors, The University of Tokyo, 2-11-16 Yayoi, Bunkyo, Tokyo 113-8656 Japan

**Keywords:** Artificial photosynthesis, Photocatalysis, Photocatalysis

## Abstract

Artificial photosynthesis of H_2_O_2_ using earth-abundant water and oxygen is a promising approach to achieve scalable and cost-effective solar fuel production. Recent studies on this topic have made significant progress, yet are mainly focused on using  organic polymers. This set of photocatalysts is susceptible to potent oxidants (e.g. hydroxyl radical) that are inevitably formed during H_2_O_2_ generation. Here, we report an inorganic Mo-doped faceted BiVO_4_ (Mo:BiVO_4_) system that is resistant to radical oxidation and exhibits a high overall H_2_O_2_ photosynthesis efficiency among inorganic photocatalysts, with an apparent quantum yield of 1.2% and a solar-to-chemical conversion efficiency of 0.29% at full spectrum, as well as an apparent quantum yield of 5.8% at 420 nm. The surface-reaction kinetics and selectivity of Mo:BiVO_4_ were tuned by precisely loading CoO_*x*_ and Pd on {110} and {010} facets, respectively. Time-resolved spectroscopic investigations of photocarriers suggest that depositing select cocatalysts on distinct facet tailored the interfacial energetics between {110} and {010} facets and enhanced charge separation in Mo:BiVO_4_, therefore overcoming a key challenge in developing efficient inorganic photocatalysts. The promising H_2_O_2_ generation efficiency achieved by delicate design of catalyst spatial and electronic structures sheds light on applying robust inorganic particulate photocatalysts to artificial photosynthesis of H_2_O_2_.

## Abstract (web summary)

An inorganic and robust photocatalytic system based on Mo-doped faceted BiVO4 particles exhibits a solar-to-chemical conversion efficiency of 0.29% for H_2_O_2_ generation, a new record among inorganic systems.

## Introduction

Harvesting solar fuels by artificial photosynthesis has great values in the global missions on tackling climate change and environmental pollutions^1–3^. Among various artificial photosynthetic reactions, solar-driven water splitting for hydrogen generation has attracted the most attention in the past half century. Yet, its practical application is challenged by the low-energy density, storability, and transportability of hydrogen gas^4,5^. To this end, photosynthesis of H_2_O_2_, an emerging liquid fuel and also a green oxidant, is attracting growing interests^6^. Among primary photosynthetic systems, including photovoltaic-assisted electrolysis^7^, photoelectrochemical catalysis^8,9^, and particulate photocatalysis (PC)^10^, PC is the most cost-effective because of its simplicity and scalability^11^. With regard to reaction processes, PC systems are advantageous for the mass transport of reagents and products, which greatly reduces the concentration overpotential and pH gradient during reactions^12^. For these reasons, it is desirable to develop efficient PC systems for H_2_O_2_ generation.

Recently, various PC systems based on organic-polymer semiconductors have been developed for photocatalytic H_2_O_2_ generation with a recording solar-to-H_2_O_2_ (STH) conversion efficiency of 0.61%^13–15^. Nevertheless, these semiconductors have a potential concern of their stability since photocatalytic H_2_O_2_ generation is inevitably accompanied by hydroxyl radical (•OH) generation (H_2_O_2_ + *hν* → 2•OH or H_2_O_2_ + e^−^ + H^+^ → •OH + H_2_O) and such a potent oxidant (*E*^0^ (•OH/H_2_O) = 2.18 V vs. NHE at pH 7.0) is damaging to organic structures^16^. For instance, after 24-h incubation under •OH-rich conditions, graphitic carbon nitride (C_3_N_4_, one of the most widely studied organic photocatalysts for H_2_O_2_ photosynthesis) lost over 60% activity for H_2_O_2_ generation (Fig. S1). In contrast, inorganic semiconductors (e.g., BiVO_4_) are resistant to •OH-mediated oxidation, so they are more favored by long-term reactions. Yet, inorganic semiconductors remain inefficient for photocatalytic H_2_O_2_ generation (<150 µM/h, see Table S1) due to high-charge recombination^13,17^. For an efficient inorganic photocatalyst, it needs to exhibit (i) a suitable band structure for O_2_ reduction and H_2_O oxidation coupled with a narrow band-gap, (ii) efficient charge separation, and (iii) high surface-reaction kinetics and selectivity.

**Fig. 1:**
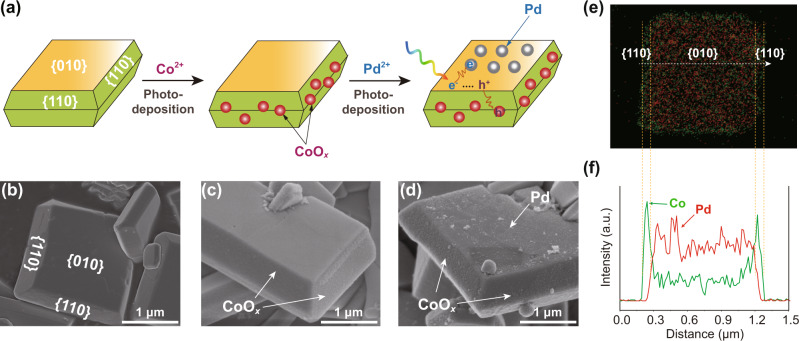
Facet-selective loading of CoO_*x*_ and Pd cocatalysts on Mo:BiVO_4_. **a** Schematic deposition processes of CoO_*x*_ and Pd on Mo:BiVO_4_ and the corresponding SEM images of **b** Mo:BiVO_4_, **c** CoO_*x*_/Mo:BiVO_4_, and **d** CoO_*x*_/Mo:BiVO_4_/Pd. **e**, **f** Energy-dispersive X-ray spectroscopy (EDS) elemental mapping and line profile along with the white arrow of CoO_*x*_/Mo:BiVO_4_/Pd.

Here, we report a faceted Mo-doped BiVO_4_ (Mo:BiVO_4_) with dual cocatalysts selectively loaded on its reduction and oxidation facets (Fig. 1a). BiVO_4_ is a photocatalyst with a suitable band structure and relatively narrow band-gap (2.4 eV) for H_2_O_2_ photosynthesis, yet the reported efficiency remains unsatisfying (<12 µM/h, see Table S1) due to severe charge recombination, even in the presence of sacrificial agents^18,19^. We synthesized monoclinic Mo:BiVO_4_ and anchored CoO_*x*_ onto the oxidative {110} facet via photooxidation, which served to promote the water oxidation kinetics. In the meantime, Pd was anchored onto the reductive {010} facet via photoreduction and served to steer the oxygen-reduction pathway from four-electron processes for H_2_O formation to two-electron processes for H_2_O_2_ generation. In-depth time-resolved spectroscopic investigations of photocarriers demonstrates that CoO_*x*_ and Pd depositions tailored the energetics of the respective facet for improved charge separation, a key obstacle limiting the performance of inorganic photocatalysts. Without using any sacrificial reagent, the reasonably designed CoO_*x*_/Mo:BiVO_4_/Pd produced H_2_O_2_ at a rate of 1425 μM/h, an apparent quantum yield (AQY) of 1.2% over the full spectrum of sunlight, and a STH of 0.29%, surpassing other inorganic photocatalysts by one order of magnitude (Table S1). With comparable efficiency with organic ones in photocatalytic H_2_O_2_ generation, our work demonstrates the feasibility of applying robust inorganic particulate photocatalysts to efficient photocatalytic H_2_O_2_ generation through delicate design of catalyst spatial and electronic structures.

## Results and discussion

### Synthesis and characterization of CoO_*x*_/Mo:BiVO_4_/Pd

We first prepared faceted Mo:BiVO_4_ particles using a solid-liquid-reaction method^19,20^. Mo was doped to the V site to increase the bulk conductivity. Mo doping amount was optimized to be 0.025 mol% based on the activity of photocatalytic H_2_O_2_ generation over CoO_*x*_/Mo:BiVO_4_/Pd (Fig. S2). The X-ray diffraction (XRD) pattern of Mo:BiVO_4_ as well as CoO_*x*_/Mo:BiVO_4_, Mo:BiVO_4_/Pd and CoO_*x*_/Mo:BiVO_4_/Pd particles matched well with that of monoclinic BiVO_4_, with {010} and {110} facet peaks located at 30.6° and 18.7°, respectively (Fig. S3). The Brunner−Emmet−Teller (BET) tests show Mo:BiVO_4_ as well as CoO_*x*_/Mo:BiVO_4_, Mo:BiVO_4_/Pd and CoO_*x*_/Mo:BiVO_4_/Pd particles exhibit similar surface areas (1.43–1.71 m^2^/g, Table S2). The Mo:BiVO_4_ particles exhibit a decahedron structure with clear facets as shown in scanning electron microscope (SEM) images (Fig. 1a, b). The selected area electron diffraction (SEAD, Fig. S4) pattern confirms the Millier index of top and side facets are {010} reduction facet and {110} oxidation facet, respectively^21^. The Mo doping amount (Mo/V) is 0.023 mol% tested by inductively coupled plasma mass spectrometry (ICP-MS).

Secondly, we selectively loaded cocatalysts onto different facets of Mo:BiVO_4_ particles via stepwise photodeposition (Fig. 1a). CoO_*x*_ as a cocatalyst for water oxidation reaction (WOR) was selectively deposited onto the {110} facet of Mo:BiVO_4_ via photooxidation of Co^2+^ ions. The loading amount of Co was optimized to be 0.2 wt% based on the activities of photocatalytic H_2_O_2_ generation over CoO_*x*_/Mo:BiVO_4_/Pd (Fig. S5). Prominent Co 2p X-ray photoelectron spectroscopy (XPS) peaks demonstrate the successful loading of Co species (Fig. S6a). The Co 2p_3/2_ peak can be deconvoluted to a Co^2+^ peak at 781.6 eV and a Co^3+^ peak at 780.6 eV, suggesting that the valence of Co was in-between +2 and +3, therefore the cocatalyst is denoted as CoO_*x*_. The SEM image (Fig. 1c) shows that CoO_*x*_ particles are uniformly distributed across the {110} facet of Mo:BiVO_4_. Consistent with the SEM results, energy-dispersive X-ray spectroscopy (EDS) elemental mapping and line profile (Fig. 1e, f) show 4.1-fold stronger Co signal on the {110} facet compared to that on the {010} facet. Further, SEM (Fig. S7) and TEM (Fig. S8) line profiles of CoO_*x*_/Mo:BiVO_4_ indicate that Co signal on {110} facet is much higher than that on {010} facet. These results demonstrate the selective deposition of CoO_*x*_ on the {110} facet of Mo:BiVO_4_.

Loading CoO_*x*_ cocatalyst significantly enhanced the WOR surface kinetics of Mo:BiVO_4_. The photocatalytic O_2_ evolution activity of CoO_*x*_/Mo:BiVO_4_ was more than twice as much as that of Mo:BiVO_4_ (Fig. S9a). The enhancement water oxidation was further verified by the improved photoelectrochemical performance of CoO_*x*_/Mo:BiVO_4_ electrode compared to that of Mo:BiVO_4_ electrode. The onset potential of the CoO_*x*_/Mo:BiVO_4_ photoanode was ~0.1 V more negative than that of Mo:BiVO_4_ (Fig. S9b). At a given potential, the photoanodic current density of CoO_*x*_/Mo:BiVO_4_ was much higher than that of Mo:BiVO_4_ (Fig. S9b). Such stark contrasts between O_2_ production and photoelectrochemical performance clearly demonstrate the improved WOR activity of Mo:BiVO_4_ upon CoO_*x*_ deposition.

Following the photodeposition of CoO_*x*_, Pd as an oxygen-reduction reaction (ORR) cocatalyst was selectively deposited on the {010} facet of Mo:BiVO_4_ via photoreduction of PdCl_4_^2−^ (Fig. 1a). The loading amount of Pd was optimized to be 0.4 wt% based on the activity of photocatalytic H_2_O_2_ generation over CoO_*x*_/Mo:BiVO_4_/Pd (Fig. S10). The distinct Pd 3d XPS peaks indicate the successful loading of Pd species (Fig. S6b). The Pd 3d_5/2_ peak can be deconvoluted to a main Pd^0^ peak at 335.1 eV and a minor Pd^2+^ peak at 337.0 eV, attributing to the metallic Pd from photoreduction and PdO from partial oxidation of Pd in air, respectively. SEM images (Fig. 1d and S11) show that Pd particles were uniformly and selectively distributed across the {010} facets of Mo:BiVO_4_. The facet-selective loading of Pd was further demonstrated by the stark contrast between distinctive Pd signal on the {010} facet and negligible Pd signal on the {110} facet from EDS elemental mapping and line profile of Mo:BiVO_4_ (Fig. 1e, f and S12). SEM (Fig. S13) and TEM (Fig. S14) line profile of Mo:BiVO_4_/Pd also suggest that Pd signal on {010} facet is much higher than that on {110} facet.

Loading Pd cocatalyst significantly enhanced the selectivity of H_2_O_2_ generation from 13% by pristine Mo:BiVO_4_ to 89% (Fig. S15; H_2_O_2_ generation selectivity is defined as the ratio of electrons utilized for H_2_O_2_ synthesis to the total number of electrons consumed). These results indicate that Pd, as a verified catalyst for selective H_2_O_2_ synthesis^22–24^, steered the ORR on Mo:BiVO_4_ from four-electron (O_2_ + 4H^+^ + 4e^−^ → 2H_2_O) to two-electron (O_2_ + 2H^+^ + 2e^−^ → H_2_O_2_) processes. Moreover, the enhanced H_2_O_2_ production selectivity with Pd loading was slightly perturbed by the presence of CoO_*x*_, likely attributed to improved H_2_O_2_ decomposition (Fig. S15). Note that the two-electron H_2_ evolution (2H^+^ + 2e^−^ → H_2_), another major side reaction likely limiting the selectivity for H_2_O_2_ generation^19^, was prohibited because the conduction band of Mo:BiVO_4_ is too deep to evolve H_2_.

### Photocatalytic performance

**Fig. 2:**
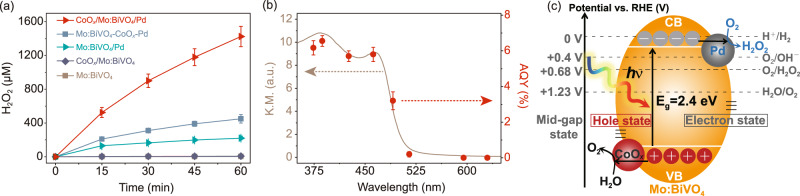
Photocatalytic H_2_O_2_ generation activities. **a** Time courses of photocatalytic H_2_O_2_ generation over CoO_*x*_/Mo:BiVO_4_/Pd, Mo:BiVO_4_-CoO_*x*_-Pd, CoO_*x*_/Mo:BiVO_4_, Mo:BiVO_4_/Pd, and Mo:BiVO_4_. Reaction conditions: photocatalyst amount, 2 mg; reactant solution, 12 mL PBS aqueous solution (pH = 7.4) saturated with O_2_; light source, xenon lamp solar simulator, 100 mW/cm^2^, AM 1.5 G. We note the data series for CoO_*x*_/Mo:BiVO_4_ (7.5 μM at the reaction time of 60 min) overlap with those of Mo:BiVO_4_ (4.1 μM at the reaction time of 60 min). **b** Apparent quantum yield (AQY) of photocatalytic H_2_O_2_ generation over CoO_*x*_/Mo:BiVO_4_/Pd as a function of the incident light wavelength. Reaction conditions: photocatalyst amount, 2 mg; reactant solution, 5 mL PBS aqueous solution (pH = 7.4) saturated with O_2_; light source, monochromatic LED light. **c** Schematic of photocatalytic H_2_O_2_ generation over CoO_*x*_/Mo:BiVO_4_/Pd. CB and VB are short for conduction band and valence band, respectively.

The photocatalytic H_2_O_2_ generation performance of the particulate photocatalyst was evaluated under simulated sunlight irradiation without any sacrificial reagent (Fig. 2a). Bare Mo:BiVO_4_ exhibits minimal performance for 60-min H_2_O_2_ generation (4.1 μM). Loading CoO_*x*_ cocatalyst enhanced the photocatalytic H_2_O_2_ generation performance by a factor of 1.8 (7.5 μM, Fig. 2a), attributing to promoted water oxidation and consequentially reduced detrimental charge recombination (Fig. 2c). In the meanwhile, loading Pd cocatalyst onto Mo:BiVO_4_ improved H_2_O_2_ generation selectivity (Fig. 2c), resulting in a 53.7-fold enhancement of 60-min H_2_O_2_ generation (220.0 μM, Fig. 2a).

Simultaneous loading of CoO_*x*_ and Pd cocatalysts enhanced H_2_O_2_ generation by 347.6-fold compared to pristine Mo:BiVO_4_ (Fig. 2a). Without any sacrificial reagent, CoO_*x*_/Mo:BiVO_4_/Pd generated 1425 µM H_2_O_2_ after one-hour reaction. When the suspension was N_2_-purged, the H_2_O_2_ production was inhibited by over 99% (Fig. S16), confirming that H_2_O_2_ generation proceeded mainly through ORR. The wavelength-dependent AQYs measured by light-emitting diode (LED) light irradiation agree well with the absorption spectrum of CoO_*x*_/Mo:BiVO_4_/Pd (Fig. 2b), suggesting that H_2_O_2_ was generated following its band-gap excitation. The AQY at 420 nm was determined to be 5.8%, the highest reported for inorganic semiconductors to the best of our knowledge (see Table S1). Furthermore, the AQY of CoO_*x*_/Mo:BiVO_4_/Pd reached 1.2% over the full spectrum, and its STH reached 0.29%. Such an efficiency surpasses other inorganic semiconductor photocatalysts by one order of magnitude (Table S1) and indicates that inorganic photocatalysts are competent for efficient photocatalytic H_2_O_2_ generation. Most importantly, in stark contrast to the high •OH-susceptibility of organic photocatalyst, after 24-h incubation under •OH-rich conditions, CoO_*x*_/Mo:BiVO_4_/Pd exhibits nominal change in H_2_O_2_ productions (Fig. S1b) and chemical compositions (Fig. S17), demonstrating its high resistance to •OH-mediated oxidation.

The photocatalytic H_2_O_2_ generation activity of CoO_*x*_/Mo:BiVO_4_/Pd decreased gradually as shown in Fig. 2a. The reaction rate in the fourth 15 min corresponds to 47% of that in the first one. To clarify such decay, CoO_*x*_/Mo:BiVO_4_/Pd was tested with cycles of reaction. The photocatalytic H_2_O_2_ generation rate in the second cycle was 420 μM/h, 29% of that in the first cycle (Fig. S18a). It is supposed that the decay is related to the gradual transformation of CoO_*x*_ to CoPi in PO_4_^3-^ solution (applied as H_2_O_2_ stabilizer^25^). Although CoPi has been widely applied to photoanodes a cocatalyst, its roles in photocatalysis are controversial mostly because of inconsistent sample conditions^26^. Here we believe that CoPi solely facilitated the surface kinetics for O_2_ evolution and hardly affected the energetics for charge separation^27^. Since the photocatalytic H_2_O_2_ generation activity is determined by the charge-separation efficiency, CoPi behaved inferior to CoO_*x*_ in our system. The transformation of CoO_*x*_ to CoPi was confirmed by characterizing CoO_*x*_/Mo:BiVO_4_/Pd before and after the reaction, where a prominent peak at 133.6 eV attributing to Co-Pi bond was observed in the post-catalysis P 2p_3/2_ XPS spectra (Fig. S19). Furthermore, the photocatalytic H_2_O_2_ generation activity of freshly prepared CoPi/Mo:BiVO_4_/Pd was 21% of that of CoO_*x*_/Mo:BiVO_4_/Pd and was similar to that of spent CoO_*x*_/Mo:BiVO_4_/Pd (Fig. S20). These results confirm that the transformation of CoO_*x*_ to CoPi undermined CoO_*x*_/Mo:BiVO_4_/Pd for photocatalytic H_2_O_2_ generation. In order to avoid the deterioration of CoO_*x*_/Mo:BiVO_4_/Pd in PO_4_^3−^ solution, photocatalytic H_2_O_2_ generation was conducted in pure water as shown in Fig. S18b. As expected, CoO_*x*_/Mo:BiVO_4_/Pd was highly stable over five cycles of reaction. To further demonstrate the stability of CoO_*x*_/Mo:BiVO_4_/Pd, we test the performance of CoO_*x*_/Mo:BiVO_4_/Pd in seawater, which is a desirable solution condition for artificial photosynthesis^28^. No deactivation was observed over five-round repetitive use (Fig. S21). Yet the cumulative production of H_2_O_2_ was lower than that in phosphate solution owing to H_2_O_2_ decomposition, consistent with the previous report^29^. In following studies, the cumulative production of H_2_O_2_ in pure water will be improved with rapid H_2_O_2_ diffusion by a large-scale photosynthesis setup where the CoO_*x*_/Mo:BiVO_4_/Pd photocatalyst will be immobilized on a support using drop-casting or screen printing technologies and integrated in a flow cell photolysis system^19,30^.

### Charge separation

We note that the enhancement of H_2_O_2_ production by the synergistic effect of coloading Pd and CoO_*x*_ is even higher than the multiplication of the enhancements by loading Pd and CoO_*x*_ individually, i.e., 347.6-fold for coloading Pd and CoO_*x*_, 53.7-fold for solely loading Pd, and 1.8-fold for solely loading CoO_*x*_. In the meantime, when CoO_*x*_ and Pd were randomly deposited on Mo:BiVO_4_ (denoted as Mo:BiVO_4_-CoO_*x*_-Pd with a SEM image in Fig. S22), it was only 32% as active as CoO_*x*_/Mo:BiVO_4_/Pd, though these two catalysts had similar surface kinetics and selectivity. Further, the photocatalytic O_2_ evolution activity of CoO_*x*_/Mo:BiVO_4_/Pd was 3.2-fold higher than that of CoO_*x*_/Mo:BiVO_4_ (Fig. S9a). These results suggest that selective coloading of CoO_*x*_ and Pd not only improved surface kinetics for O_2_ evolution and selectivity for H_2_O_2_ production as introduced above, but also tuned other critical processes like charge separation^31^. Furthermore, selectively coloading dual cocatalysts has been applied to enhance charge separation for sacrificial photocatalytic O_2_ evolution^21,32,33^. To this end, charge separation processes in Mo:BiVO_4_, CoO_*x*_/Mo:BiVO_4_, Pd/Mo:BiVO_4_, CoO_*x*_/Mo:BiVO_4_/Pd were thoroughly studied by transient absorption spectroscopy (TAS).

**Fig. 3:**
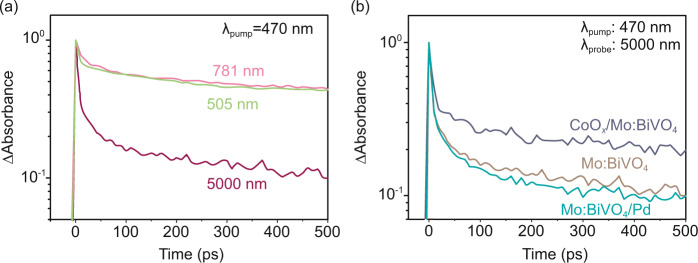
Charge-carrier dynamics at picosecond time-scale. **a** Transient profiles of photocarriers probed at 505 nm (trapped holes), 781 nm (deeply trapped electrons), and 5000 nm (free/shallowly trapped electrons) in Mo:BiVO_4_. **b** Transient profiles of photocarriers probed at 5000 nm (free/shallowly trapped electrons) for Mo:BiVO_4_, CoO_*x*_/Mo:BiVO_4_ and Mo:BiVO_4_/Pd. Pump wavelength: 470 nm (4 μJ pulse^−1^).

The TA spectra of photogenerated charge carriers in Mo:BiVO_4_ was examined upon band-gap excitation as shown in Fig. S23. The spectra exhibited strong absorption in 20,000–17,000 cm^−1^, a broad absorption in 170,000–5000 cm^−1^, and a weak absorption <5000 cm^−1^. These absorptions are attributed to trapped holes, deeply trapped electrons and free/shallowly trapped electrons, respectively (see supplementary discussions in Figs. S23 for detailed peak assignments)^34–36^. The dynamics of these photocarriers were compared in the ultrafast region (picosecond time-scale) as shown in Fig. 3. The photocarriers probed at 781 nm (~1.59 eV, deeply trapped electrons) and 505 nm (~2.45 eV, trapped holes) exhibited comparably slow decay kinetics, suggesting that these photocarriers recombined non-radiatively. In contrast, the photocarriers probed at 5000 nm (the free/shallowly trapped electrons) exhibited much faster decay kinetics, which well explains the weak absorption <5000 cm^−1^ shown in Fig. S23. This result indicates that the free/shallowly trapped electrons were rapidly trapped by the mid-gap states in Mo:BiVO_4_, consistent with previous studies^35,36^. Regardless of such an unfavorable effect, the TA signals for this kind of photocarriers were still observed to vary significantly after loading Pd or CoO_*x*_ on Mo:BiVO_4_ (Fig. 3b). Loading Pd on the {010} facet of Mo:BiVO_4_ accelerated the decay of the free/shallowly trapped electrons since Pd captured electrons. In contrast, loading CoO_*x*_ on the {110} facet lead to an opposite effect because CoO_*x*_ captured holes and thus increased the electron population in Mo:BiVO_4_. At 50 ps, for instance, loading CoO_*x*_ increased the intensity of TA signal for free/shallowly trapped electrons by ~66%, while loading Pd only decrease the intensity by ~6% compared to that of bare sample. The effect of CoO_*x*_ on the dynamics of the free/shallowly trapped electrons was more intense than that of Pd because electron transfer to Pd competed with trapping of electrons into deep trap states below the CB. Electron trapping to deep trap states was represented by the strong TA signal probed at 781 nm as shown in Fig. 3a.

**Fig. 4:**
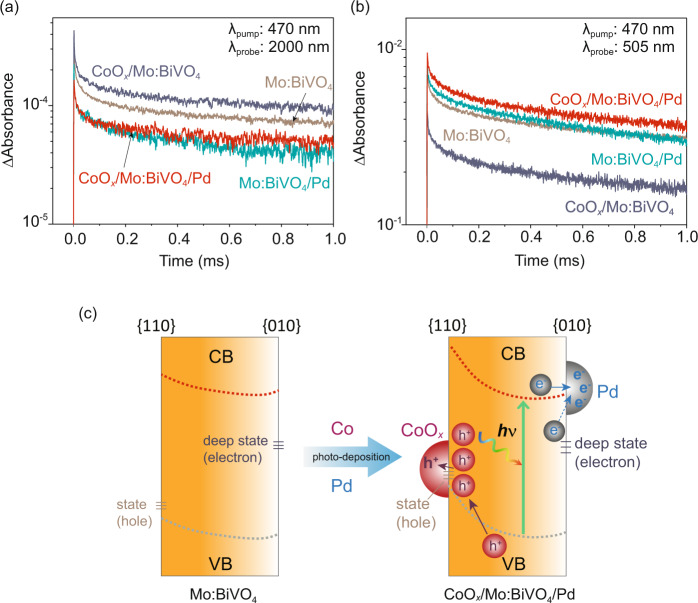
Charge-carrier dynamics at microsecond time-scale and impacts of cocatalysts on the energetics of Mo:BiVO_4_ facets. Transient profiles of photocarriers probed **a** at 2000 nm (free/shallowly trapped electrons) and **b** 505 nm (trapped holes) for Mo:BiVO_4_, CoO_*x*_/Mo:BiVO_4_, Mo:BiVO_4_/Pd, and CoO_*x*_/Mo:BiVO_4_/Pd. Samples were excited by 470 nm laser pulses (Surelite I, duration: 6 ns, fluence: 3 mJ pulse^−1^, repetition: 1 Hz). **c** Impacts of cocatalysts on the energetics of Mo:BiVO_4_ facets.

Considering the crucial impact of electron trapping on electron-transfer processes in the picosecond region, the electron dynamics was further examined in the microsecond-millisecond region where photoexcited electrons in Mo:BiVO_4_ are expected to have already relaxed in the trap states. As shown in Fig. 4a, Pd and CoO_*x*_ accelerated and decelerated the decay of free/shallowly trapped electrons, respectively, similar to the effects in picosecond region (Fig. 3b). This result indicates that in this time region, Pd and CoO_*x*_ captured electrons and holes, respectively, as expected. The population of free/shallowly trapped electrons that remained in Mo:BiVO_4_ after electrons are captured by Pd can be determined by estimating the ratio of ΔAbsorbance displayed in Fig. 4a with respect to bare Mo:BiVO_4_ and CoO_*x*_/Mo:BiVO_4_ for Mo:BiVO_4_/Pd and CoO_x_/Mo:BiVO_4_/Pd, respectively. At 200 μs, for instance, 59% and 42% of free/shallowly trapped electrons remained in Mo:BiVO_4_ for Mo:BiVO_4_/Pd and CoO_*x*_/Mo:BiVO_4_/Pd, respectively. This implies that 41 and 58% of free/shallowly trapped electrons transferred to Pd for Mo:BiVO_4_/Pd and CoO_*x*_/Mo:BiVO_4_/Pd, respectively. More electrons transferred to Pd on CoO_*x*_/Mo:BiVO_4_/Pd than on Mo:BiVO_4_/Pd because of more efficient charge separation (synergistic charge separation) in the former case. To further justify this effect, the impact of CoO_*x*_ and Pd on the decay kinetics of trapped holes in Mo:BiVO_4_ was investigated. To further justify this effect, the impact of CoO_*x*_ and Pd on the decay kinetics of trapped holes in Mo:BiVO_4_ was investigated. As depicted in Fig. 4b, CoO_*x*_ and Pd accelerated and decelerated the decay of accumulated trapped holes, respectively. Surprisingly, in CoO_*x*_/Mo:BiVO_4_/Pd, the effect of CoO_*x*_ on capturing photogenerated holes in Mo:BiVO_4_ was compensated by the effect of Pd on accumulating trapped holes by efficiently trapping electrons. Furthermore, CoO_*x*_/Mo:BiVO_4_/Pd accumulated more trapped holes in Mo:BiVO_4_, even higher than that in Mo:BiVO_4_/Pd. This finding reveals that electrons and holes were efficiently separated at different facets. Aside from accumulating photogenerated holes in Mo:BiVO_4_ and facilitating the transfer of free/shallowly trapped electrons to Pd, loading cocatalysts also activated the deeply trapped electrons in Mo:BiVO_4_ for photocatalysis. As shown in Fig. S24, the TA signal and decay kinetics of the deeply trapped electrons in CoO_*x*_/Mo:BiVO_4_/Pd were similar to that of Pd/Mo:BiVO_4_ and apparently different from that of Mo:BiVO_4_ and CoO_*x*_/Mo:BiVO_4_. This result suggests that the deeply trapped electrons transferred to Pd and became available for subsequent surface reactions in Mo:BiVO_4_/Pd and CoO_*x*_/Mo:BiVO_4_/Pd. The charge transfer involving deeply trapped electrons to Pd cocatalyst is possible via tunneling and trap-to-trap hopping. A similar diffusion of trapped electrons is often proposed to take place on long-lived persistent phosphor materials^37^, wherein the trap states are situated at ~0.5–1.0 eV below the conduction band minimum. Aside from this diffusion process, re-excitation of deeply trapped electrons to the CB and eventually transfer to the Pd will be possible. Taken together, the above findings demonstrate that selectively coloading Pd and CoO_*x*_ on the expected facets of Mo:BiVO_4_ significantly enhanced the charge separation and suppressed rapid charge-carrier trapping and recombination. Such positive effects are supposed to be achieved by tuning the energetics between cocatalysts and respective Mo:BiVO_4_ facet (Fig. 4c and S25).

In conclusion, we developed an inorganic semiconductor-based system for efficient overall photocatalytic H_2_O_2_ generation. Faceted Mo:BiVO_4_ particles was used as a light absorber and its {110} and {010} facets were selectively loaded with CoO_*x*_ and Pd as WOR and ORR cocatalysts, respectively. These cocatalysts in such a configuration greatly improved the kinetics and selectivity for surface reactions. Furthermore, the spatial separation of cocatalysts on different facets of Mo:BiVO_4_ significantly enhanced the charge separation and suppressed rapid charge-carrier trapping and recombination, a key challenge in improving the efficiency of inorganic photocatalysts. With these merits, CoO_*x*_/Mo:BiVO_4_/Pd generated H_2_O_2_ with an AQY of 1.2% at full spectrum and a STH of 0.29%, a new record for inorganic semiconductor-based systems.

## Methods

### Catalyst preparation

Single crystal Mo:BiVO_4_ was prepared by heating the mixture of K_2_CO_3_ (1.047 g), MoO_3_ (1.8 mg) and V_2_O_5_ (2.272 g) in a ceramic crucible at a heating rate of 1.5 °C/min to 450 °C and annealing for 5 h in a muffle furnace. The obtained Mo:K_3_V_5_O_14_ (2 g) was mixed with Bi(NO_3_)_3_•5H_2_O (0.326 g) and dispersed in 50 mL deionized water under ultrasonication for 30 min. The mixture was stirred and heated at 70 °C for 10 h under ultrasonication, separated by centrifugation, washed with deionized water, and dried at 70 °C for 8 h. As-prepared Mo:BiVO_4_ (0.2 g) was dispersed in 100 mL water, followed by addition of 0.1 mol NaIO_3_ and 0.27 mL Co(NO_3_)_2_ stock (1.5 g/L). The mixture was irradiated at *λ* > 420 nm for 3 h using a xenon lamp solar simulator (model 300 DUV; Perfect Light, Inc., light intensity = 0.1 W/cm^2^), filtered, washed with deionized water, and dried at 60 °C for 8 h. The as-prepared CoO_*x*_/Mo:BiVO_4_ (0.15 g) was dispersed in 100 mL pure water, followed by addition of 0.18 mL Na_2_PdCl_4_ stock (3.3 g/L). The mixture was irradiated at *λ* > 420 nm for 3 h. As-prepared CoO_*x*_/Mo:BiVO_4_/Pd was filtered, washed with deionized water, and dried at 60 °C for 8 h. Same photodeposition method was also applied in preparing Mo:BiVO_4_/Pd except for using Mo:BiVO_4_ as the starting material. Mo:BiVO_4_-CoO_*x*_-Pd was prepared following an impregnation procedure by air-purging the mixture of Mo:BiVO_4_ (0.2 g), Co(NO_3_)_2_ (0.4 mg) and Na_2_PdCl_4_ (0.8 mg) (from stock) until dry, followed by heating in a ceramic crucible at a heating rate of 5 °C/min to 200 °C and annealing for 0.5 h under reductive condition (10% H_2_ and 90% Ar) in a tube furnace. The elemental compositions were analyzed by EDS, XPS, and ICP-MS (Figs. S26 and 27 and Table S3).

### Photocatalyst characterizations

XPS measurements were performed with a Thermo Scientific 250Xi system with monochromatic Al Kα as the excitation source. The XRD patterns were recorded with a Bruker D8 Advance X-ray diffractometer with Cu K*α* radiation (*λ* = 1.5406 Å) operated at 40 kV and 40 mA. The BET tests were performed by an ASAP 2460 with N_2_ analysis adsorptive at 77.2 K. SEM images were taken using a Hitachi SU-8010 microscope equipped with EDS at 30 kV. TEM images were taken using a Hitachi 7650 microscope operated at 100 kV. UV-DRS spectra was taken with a Shimadzu UV-3600 with a resolution of 0.1 nm. The ICP-MS measurement was performed with a NexION 300X (detection limit 1 μg/L).

### Photocatalytic activity tests

Photocatalyst (24 mg) was dispersed in 12 mL deionized water with 1 M phosphate buffer (pH 7.4) in a custom-made reactor containing a quartz window. The catalyst was dispersed by ultrasonication for 10 min and purged with O_2_ for 20 min. All the equipment needed is shown in Fig. S28. Photocatalytic production of H_2_O_2_ was assessed by irradiating photocatalyst suspension using a xenon lamp solar simulator (model 300 DUV; Perfect Light Inc.) under water bath (12 ± 0.5 °C). The light intensity was adjusted to 100 mW/cm^2^ (AM 1.5 G; irradiation area = 1.83 cm^2^). The Xenon lame and the standard AM1.5 G (ASTMG 173) spectrum is shown in Fig. S29. For the wavelength-dependent AQY analysis, the photolysis was performed using LED light irradiation (model slight; Perfect Light, Inc.). At designated time points, 50 μL suspension was taken for analysis of H_2_O_2_ productions and diluted with phosphate buffer (pH = 7.4) to a H_2_O_2_ concentration (2–20 μM) that is most suitable for accurate H_2_O_2_ quantification, followed by centrifugation. Then 50 μL supernatant was taken and mixed with 50 μL solutions containing phosphate buffer (50 mM, pH = 7.4), ampliflu red (100 µM) and horseradish peroxidase (0.05 U/mL). Ampliflu red selectively reacted with H_2_O_2_ in the presence of horseradish peroxidase and formed the product resorufin. Resorufin in the mixture solution was quantified using an Agilent high-performance liquid chromatography coupled to a photo-diode array detector (detection at 560 nm); 50 µL of each sample was injected. The calibration in Fig. S30 is used to quantitatively analyze the H_2_O_2_ concentration. Separation was carried out in a C18 column at 20 °C with an isocratic mobile phase of 55% sodium citrate buffer (with 10% methanol (v/v), pH 7.4) and 45% methanol (v/v) at a flow rate of 0.5 mL min^−1^. The AQY was determined using:

1 $${{{{{\rm{AQY}}}}}}=\frac{2\times {[{{{{{{\rm{H}}}}}}}_{2}{{{{{{\rm{O}}}}}}}_{2}]}_{1{{{{{\rm{h}}}}}}}\times {{V}}}{{{{I}}}_{{{{{{{\rm{tot}}}}}}\_{{{{{\rm{p}}}}}}}}\times {{A}}\times {{t}}}$$

where [H_2_O_2_]_1h_ is the H_2_O_2_ concentration, *I*_tot_p_ is the total photo flux of simulated sunlight irradiation (4.4 × 10^−3^ mol/m^2^/s, calculation details were shown in section S3), *V* is the volume of suspension (12 mL), *A* is the irradiation area (1.91 cm^2^ in this study), and *t* is the reaction time (1 h).

The STH was determined using:

2 $${{{{{\rm{STH}}}}}}=\frac{\triangle {{G}}\left({{{{{{\rm{H}}}}}}}_{2}{{{{{{\rm{O}}}}}}}_{2}\right)\times {[{{{{{{\rm{H}}}}}}}_{2}{{{{{{\rm{O}}}}}}}_{2}]}_{1{{{{{\rm{h}}}}}}}\times {{V}}}{{{{I}}}_{{{{{{{\rm{tot}}}}}}\_{{{{{\rm{e}}}}}}}}\times {{A}}\times {{t}}}$$

Where Δ*G*(H_2_O_2_) is the free energy for H_2_O_2_ formation (117 kJ mol^–1^), *I*_tot_e_ is the total intensity of simulated sunlight irradiation (0.1 W/cm^2^), *V* is the volume of suspension (12 mL), *A* is the irradiation area (1.91 cm^2^ in this study), and *t* is the reaction time (1 h).

### Photoelectrochemical characterizations

Particle-based electrodes was prepared by a particle transfer method^38^. Firstly, 10 mg prepared CoO_*x*_/Mo:BiVO_4_ particles were suspended in a 450 μl isopropanol, followed by sonicated for 5 min. Secondly, the uniform suspension solution was dropped casting on a 1 × 3 cm glass substrate and fully dried in air. Thirdly, a thin layer of Ti (2–5 nm) was sputtered on the CoO_*x*_/Mo:BiVO_4_ particles. At last, the transferred electrode was sonicated for 10 s in water to remove the excessive particles on the surface. The electrochemical properties were assessed on a Biologic SP150 electrochemical analyzer using a three-electrode cell with the as-prepared electrode as the working electrode, Ag/AgCl as the reference electrode, and glassy carbon as the counter electrode. Cyclic voltammetry curves were obtained in a N_2_- or O_2_-saturated phosphate buffer solution (0.5 M, pH = 6.5). All potentials versus Ag/AgCl were converted to values vs. RHE.

### Photocarriers dynamics by TAS measurement

Microsecond-millisecond TAS characterizations were performed using Nd:YAG laser system (Continuum, Surelite I) equipped with custom-built spectrometers^39,40^. Briefly, the TA spectra was measured from 20,000 cm^−1^ (500 nm)–1600 cm^−1^ (6250 nm) after band-gap excitation using 470 nm laser pulses (duration: 6 ns, fluence: 3 mJ pulse^−1^). For the IR probing, the light emitted from the MoSi_2_ coil was focused on the sample and then the reflected light from the sample was introduced to a grating spectrometer. The photoexcited electrons was probed at 2000 cm^−1^ (2000 nm). The monochromated light was detected by a mercury cadmium telluride (MCT) detector (Kolmar). Meanwhile, the photogenerated trapped electrons and holes in the non-modified and (CoO_*x*_, Pd)-modified Mo:BiVO_4_ were probed at 12,800 cm^−1^ (781 nm) and 19,800 cm^−1^ (505 nm, 2.45 eV), respectively. The output electric signal was amplified with an AC-coupled amplifier (Stanford Research Systems, SR560, 1 MHz). The time resolution of the spectrometer was limited to 1 μs by the response of the MCT detector. The output electric signal was amplified using AC-coupled amplifier with a bandwidth of 1 MHz, which measures responses from one microsecond to milliseconds. Three thousand responses were accumulated to obtain the intensity trace at one particular wavenumber or probe energy. The experiments were carried out in vacuum and at room temperature.

Femtosecond time-resolved absorption measurements were performed by employing a pump-probe technique based on femtosecond Ti:Sapphire laser system (Spectra Physics, Solstice & TOPAS prime; duration = 90 fs; repetition rate = 1 kHz)^40^. The time resolution of this spectrometer was ~90 fs. Briefly, in this experiment, the photoexcited charge carriers in the photocatalysts were probed at 19,800 cm^−1^ (505 nm), 12,800 cm^−1^ (781 nm), and 2000 cm^−1^ (5000 nm). In the mid-IR absorption measurement, the probe light transmitted from the sample was detected by an MCT detector (Kolmar), while in the visible to near-infrared region, the diffuse-reflected probe light was detected by photomultiplier (Hamamatsu Photonics, H11903-20). The samples were excited by 470-nm pulses (duration: 90 fs, fluence: 4 μJ pulse^−1^). To obtain the absorbance change with a good signal-to-noise ratio, the pump pulses were chopped using an optical chopper at 500 Hz and the signal acquisition was carried out on a shot-by-shot basis at a rate of 1 kHz. The decay curves were obtained at 10 ps intervals and accumulated signals were averaged over 1000–4000 scans for one point. TAS measurements were performed in vacuum (base pressure ~ 10^−5^ Torr). For sample preparation, each Mo:BiVO_4_ and (Pd, CoO_*x*_)-loaded Mo:BiVO_4_ powders was prepared by dispersing the powder on isopropanol and then drop-casted on a circular CaF_2_ substrate and subsequently dried naturally in air to obtain a powder film with a density of ~ 1.25 mg cm^−2^.

## Supplementary information

Supplementary Information

Peer Review File

## Data Availability

Source data are provided with this paper.

## Source data

Source data
