# Supplementary material for: Overall photosynthesis of H2O2 by an inorganic semiconductor
Source: Nat Commun. 2022 Feb 24;13:1034. doi: 10.1038/s41467-022-28686-x (PMC8873311; doi:10.1038/s41467-022-28686-x)
Supplement: Supplementary file 1 — Supplementary Information [file 41467_2022_28686_MOESM1_ESM.pdf]

Supplementary Information

Overall photosynthesis of H_2O_2 by an inorganic semiconductor

Tian Liu,[1] Zhenhua Pan,[2]* Junie Jhon M. Vequizo,[3] Kosaku Kato,[4] Binbin Wu,[1] Akira Yamakata,[4] Kenji Katayama,[2] Baoliang Chen,[1] Chiheng Chu,[1]* Kazunari Domen[3,5]

[1] Faculty of Agriculture, Life, and Environmental Sciences, Zhejiang University, Hangzhou 310058, China

[2] Department of Applied Chemistry, Faculty of Science and Technology, Chuo University, 1-13-27 Kasuga, Bunkyo, Tokyo 112-8551, Japan

[3] Research Initiative for Supra-Materials, Shinshu University, 4-17-1 Wakasato, Nagano-shi, Nagano 380-8553, Japan

[4] Graduate School of Engineering, Toyota Technological Institute, 2-12-1, Hisakata, Tempaku, Nagoya 468-8511, Japan

[5] Office of University Professor, The University of Tokyo, 2-11-16 Yayoi, Bunkyo, Tokyo 113-8656, Japan

* Corresponding Author: zhenhua.20y@g.chuo-u.ac.jp, chuchiheng@zju.edu.cn

Section S1. Chemicals

All chemicals were used as received without further purification. Acetic acid (> 99.7%), hydrogen peroxide, sodium phosphate dibasic, sodium dihydrogen phosphate, methanol (HPLC grade, >99.9%), formaldehyde (37%, w/w), resorufin sodium salt, amplex red, peroxidase (from horseradish), hydrogen peroxide solution (>30%, w/w), cobalt(II) nitrate, sodium tetrachloropalladionate, ammonium metavanadate, bismuth nitrate and *N*-(3-Dimethylaminopropyl)-*N'*-ethylcarbodiimide hydrochloride were obtained from Sigma Aldrich. All solutions were prepared using ultrapure water (>18.2 MΩ•cm) produced by Millipore Milli-Q Water Purification System.

Section S2. Assessment of H_2O_2 selectivity

H_2O_2 selectivity by photocatalyst was assessed using methanol as electron donor.[1] H_2O_2 selectivity is defined as the ratio of electrons utilized for H_2O_2 synthesis to the total number of electrons consumed (i.e., electrons donated by methanol). The consumption amount of methanol was assessed by analyzing its oxidation products (formaldehyde). Experimental suspensions contained 2 g/L photocatalyst and 10% methanol (v/v). The suspension was O_2 saturated by continuous purging during the irradiation.

Nash's reagent was used for quantification of formaldehyde. Nash's reagent (0.5 mL) containing 2 M ammonia acetate, 30 mM acetyl acetone, and 35 mM acetic acid was mixed with the sample suspension at 1:1 ratio and heated in oven at 60 °C for 1 hour. The absorption spectra of products were measured using UV-Vis spectrometer at 415 nm. Formaldehyde standard solution was used to calibrate the absorption in relation to formaldehyde concentration.

Section S3. Calculation of energy- and photon-flux of solar irradiation

The total intensity of simulated sunlight irradiation (I_{tot_e}) was adjusted to 100 mW/cm^2 using a radiometer. The fraction of energy flux at each wavelength (f_{λ_e}) was calculated from the fraction of photon flux (f_{λ_p}):

$$f_{\lambda\_e} = \frac{f_{\lambda\_p}/\lambda}{\sum f_{\lambda\_p}/\lambda}$$

where λ is the wavelength of light. The energy flux at each wavelength (I_{λ_e}) was subsequently calculated, accounting for the fraction of light intensity at each wavelength.

$$I_{\lambda\_e} = I_{tot\_e} f_{\lambda\_e}$$

The photon flux at each wavelength (I_{λ_p}) was calculated by dividing the energy flux at each wavelength by the energy of a photon at each wavelength:

$$I_{\lambda\_p} = \frac{I_{\lambda\_e}}{hc/\lambda} / N_A$$

where h is the Planck's constant (6.63×10^{-34} J•s), c is the light speed (3.0×10^{8} m/s), and N_A is the Avogadro's number (6.02×10^{23}). The total photon flux (I_{tot_p}) was calculated to be 4.4×10^{-3} $mol/m^2/s$ by the sum of photon flux at each wavelength:

$$I_{tot\_p} = \sum I_{\lambda\_p}$$

Table S1. Comparison of photocatalytic H_2O_2 production.

Photocatalyst	Experimental Conditions			Rate ($\mu M\ h^{-1}$)	AQY % (420 nm)	AQY % (full spectrum)	STH %	Ref.
	Sacrificial agent	Gas	Light					
**Inorganic Photocatalyst**								
CoO_x/Mo:$BiVO_4$/Pd	No	O_2	AM 1.5	1425	5.8	1.2	0.29	This work
Pd/TiO_2	No	Air	AM 1.5	150	-	-	-	[2]
rGO/TiO_2/CoPi	No	O_2	λ >320 nm	60	-	-	-	[3]
GO	No	Air	Simulated sunlight	50	-	-	-	[4]
rGO/TiO_2/P	No	O_2	λ >320 nm	30	-	-	-	[3]
Au/$BiVO_4$	No	O_2	λ >420 nm	12	0.24	-	-	[5]
$BiVO_4$	No	O_2	λ >420 nm	<0.5	-	-	-	[5]
**Organic Photocatalyst**								
RF523	No	O_2	420-700 nm	2067	8	-	0.5	[6]
Sb-SAPC	No	O_2	AM1.5	588	17.6	-	0.61	[7]
Co_1/AQ/C_3N_4	No	O_2	AM 1.5	62	-	0.054	0.014	[1]
g-C_3N_4/BDI50	No	O_2	420-500 nm	27.8	2.6	-	0.13	[8]
g-C_3N_4/PDI/$RGO_{0.05}$	No	O_2	AM 1.5	24	6.1	-	0.2	[9]
g-C_3N_4	No	O_2	λ >420 nm	175	4.3	-	0.26	[10]
ZnPPc/NBCN	No	O_2	400-800 nm	11.4	-	-	-	[11]

Table S2. The result of BET test.

**Sample**	$Mo:BiVO_4$	$CoO_x/Mo:BiVO_4$	$Mo:BiVO_4/Pd$	$CoO_x/Mo:BiVO_4/Pd$
**BET (m^2/g)**	1.43	1.56	1.60	1.71

Table S3. The Mo/V, Pd/Bi and CoBi atomic yield of $CoO_x/Mo:BiVO_4/Pd$.

	Mo/V	Pd/Bi	Co/Bi
XPS	-	2.0%	1.7%
EDS	-	2.3%	2.2%
ICP	0.023%	0.34%	0.22%

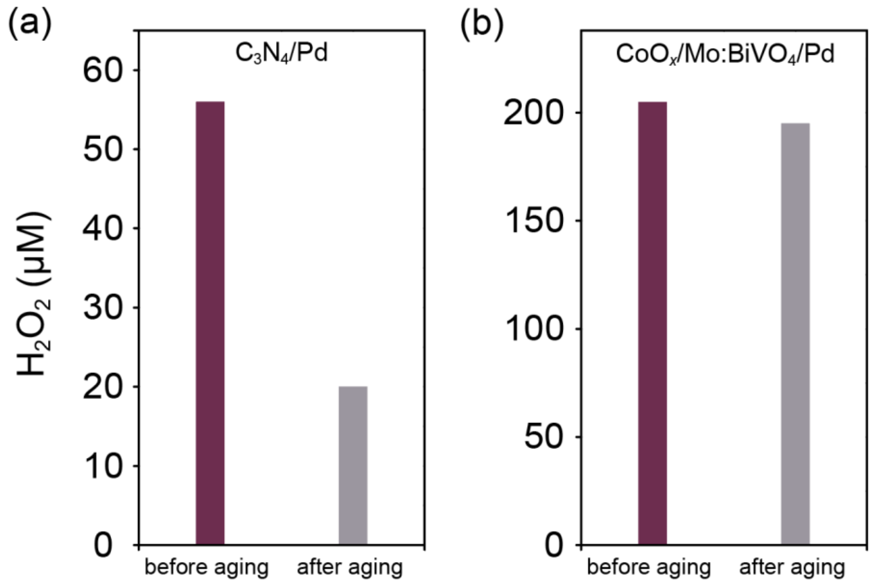

Figure S1. Photocatalytic H_2O_2 generation after one-hour reaction over (a) C_3N_4/Pd and (b) CoO_x/Mo:BiVO_4/Pd before and after •OH-aging. Aging conditions: photocatalyst amount, 2 mg; reactant solution, 25 mM H_2O_2; light source, UV-C light (254 nm); reaction time, 24 hours. Photocatalytic H_2O_2 generation conditions: photocatalyst amount, 2 mg; reactant solution, 12 ml pure water saturated with O_2; light source, xenon lamp solar simulator, 100 mW/cm^2, AM 1.5G. Bulk C_3N_4 was prepared by heating melamine powder at a heating rate of 1 °C/min to 550 °C and annealing for 5 h. As-prepared bulk C_3N_4 was exfoliated by probe sonication for 8 h, separated by centrifugation, washed with deionized water, and dried at 60 °C overnight. The C_3N_4/Pd was prepared by dispersing 0.2 g C_3N_4 and 0.24 ml Na_2PdCl_4 solution (3.3 g/L) in 50 mL pure water, followed by irradiated with a 300 W Xe lamp (⩾420 nm) under continuous stirring for 3 h, centrifuged and washed with deionized water for 3 times, and dried at 60 °C for 8 h.

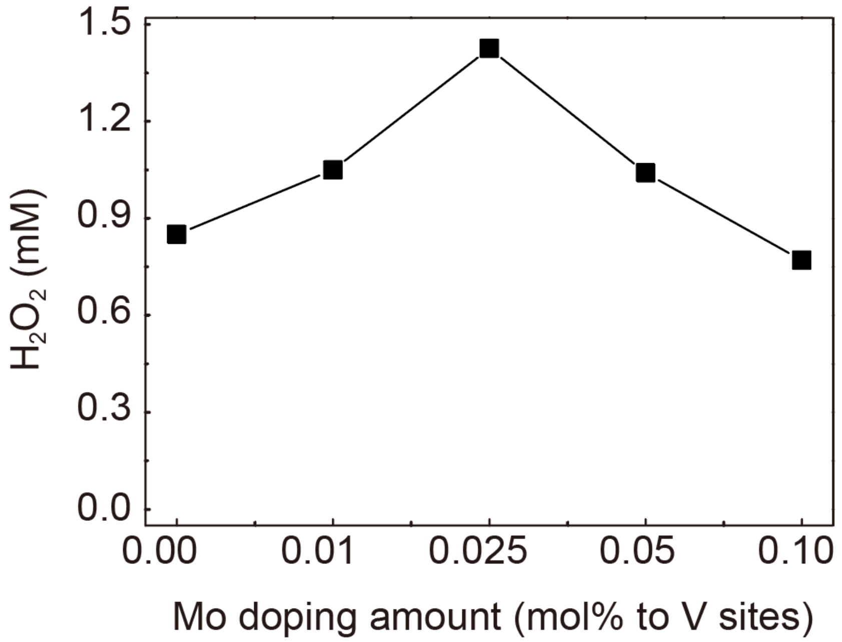

Figure S2. Amounts of H_2O_2 generation over CoO_x/Mo:$BiVO_4$/Pd as a function of the Mo doping amount. The loading amounts of CoO_x and Pd were 0.2 wt% and 0.4 wt%, respectively. Reaction conditions: photocatalyst amount, 2 mg; reactant solution, 12 ml PBS aqueous solution (pH=7.4) saturated with O_2; light source, xenon lamp solar simulator, 100 mW/cm^2, AM 1.5G.

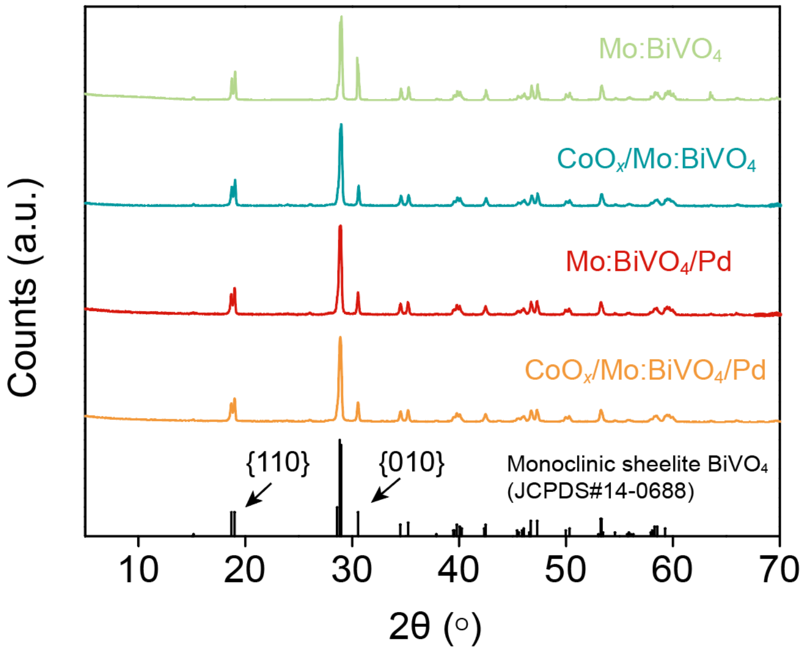

Figure S3. The XRD patterns of Mo:$BiVO_4$, CoO_x/Mo:$BiVO_4$, Mo:$BiVO_4$/Pd and CoO_x/Mo:$BiVO_4$/Pd. These XRD patterns are in good agreement with the JCPDS standard card #14-0688, corresponding to monoclinic scheelite $BiVO_4$.

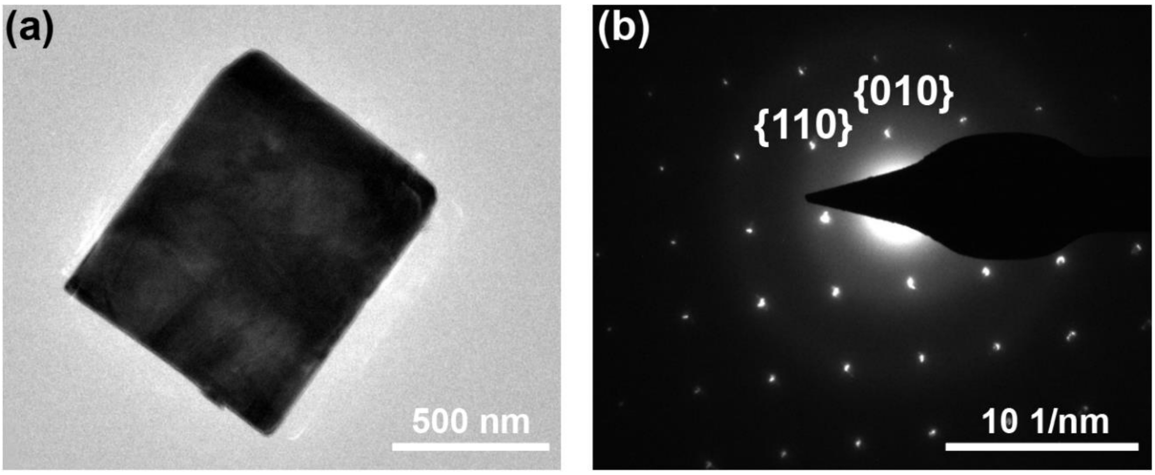

Figure S4. (a) Transmission electron microscopy image and (b) selected area electron diffraction (SAED) pattern of an as-prepared Mo:$BiVO_4$ particle.

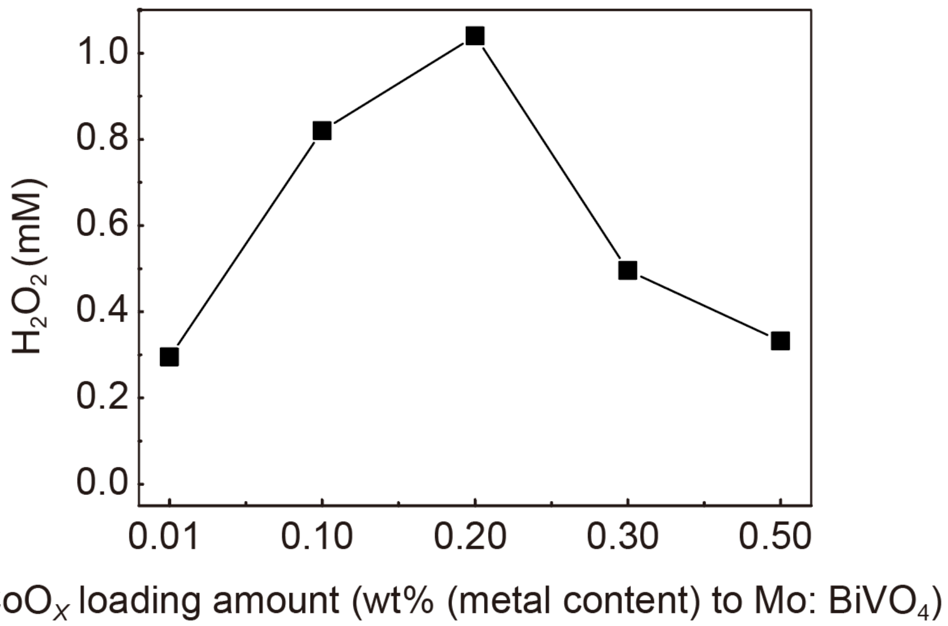

Figure S5. Amounts of H_2O_2 generation over CoO_x/Mo:$BiVO_4$/Pd as a function of the CoO_x loading amount. Reaction conditions: photocatalyst amount, 2 mg; reactant solution, 12 ml PBS aqueous solution (pH=7.4) saturated with O_2; light source, xenon lamp solar simulator, 100 mW/cm^2, AM 1.5G.

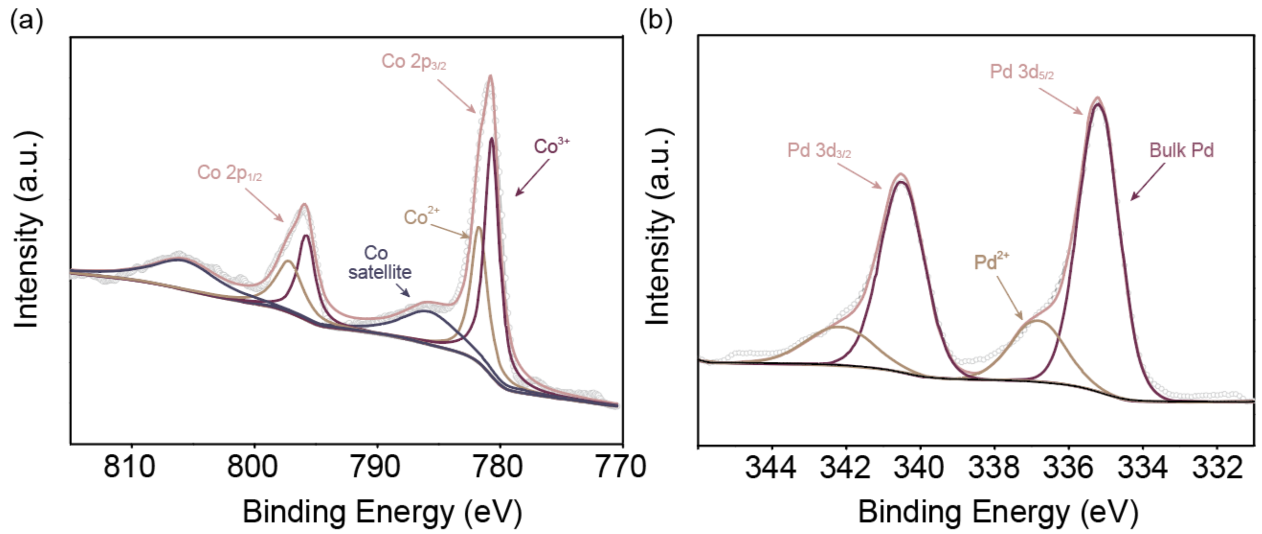

Figure S6. (a) Co 2p XPS spectra of CoO_x/Mo:$BiVO_4$. (b) Pd 3d XPS spectra of Mo:$BiVO_4$/Pd. The area of Co $2p_{3/2}$ peak is twice of that of Co $2p_{1/2}$ peak and the binding energies of Co^{3+} and Co^{2+} are 780.6 and 781.6 eV, respectively. The area of Pd $3p_{5/2}$ peak is 1.5 times of that of Pd $2p_{3/2}$ peak and the binding energies of bulk Pd and Pd^{2+} are 335.1 and 337.0 eV, respectively.

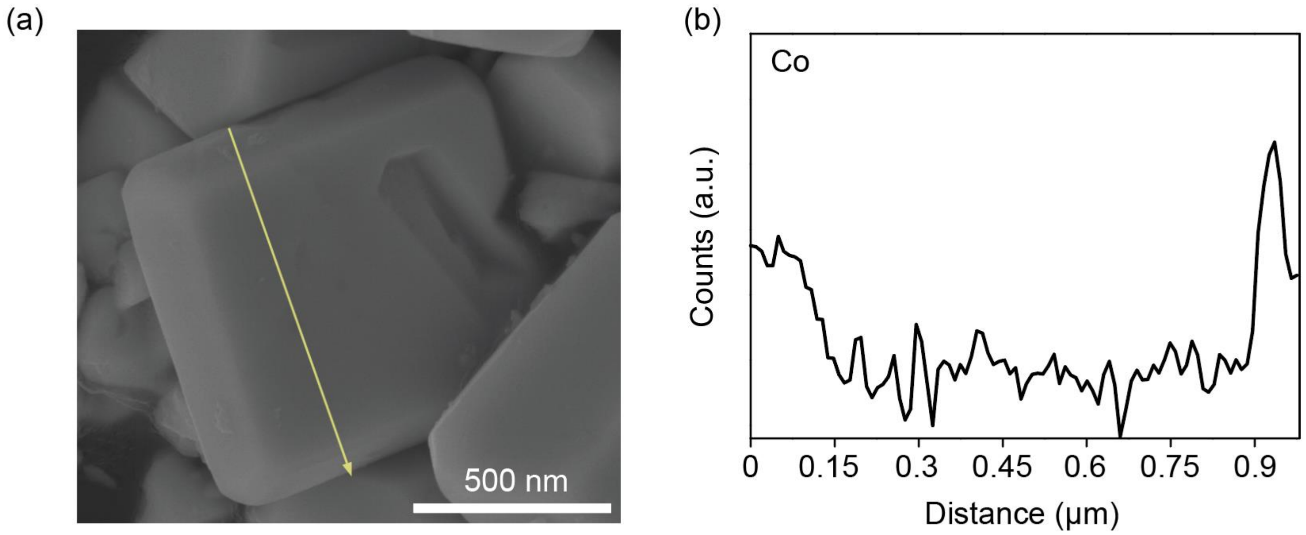

Figure S7. (a) SEM image of CoO_x/Mo:$BiVO_4$. (b) EDS line profile of Co along with the yellow arrow of CoO_x/Mo:$BiVO_4$.

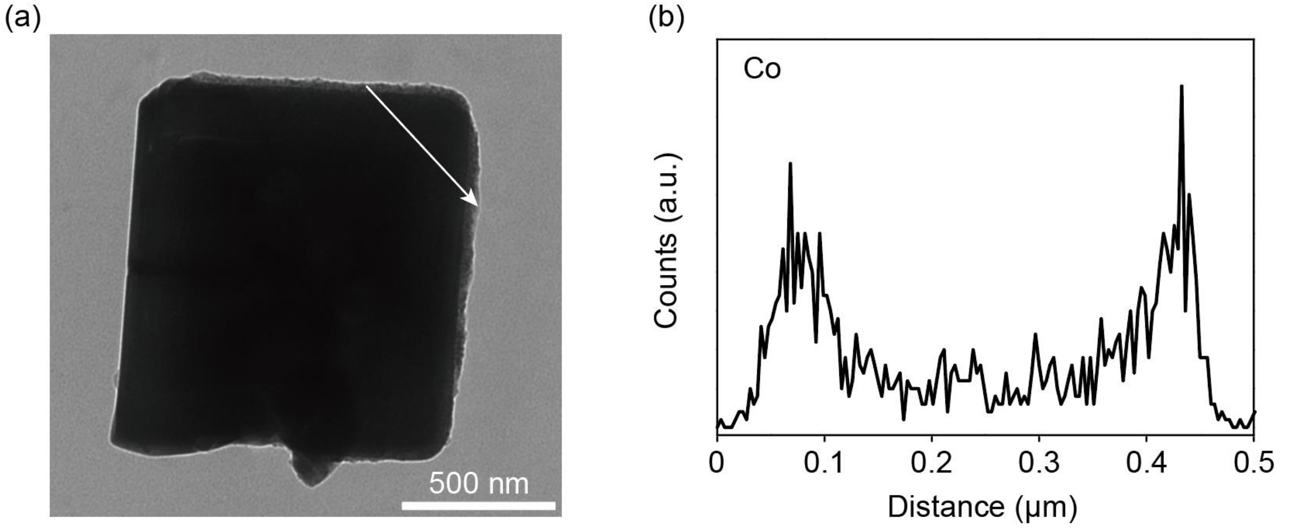

Figure S8. (a) TEM image of CoO_x/Mo:$BiVO_4$. (b) EDS line profile of Co along with the white arrow of CoO_x/Mo:$BiVO_4$.

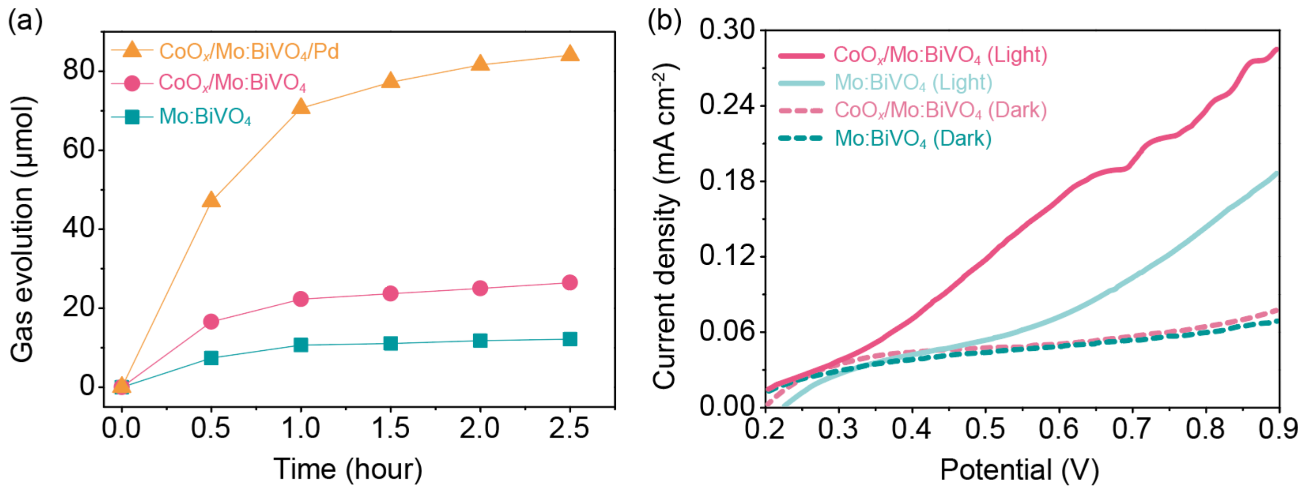

Figure S9. (a) Time course of O_2 evolution over Mo:BiVO_4 with or without CoO_x and Pd. Reaction conditions: photocatalyst amount, 0.1 g; reactant solution, 100 mL water with 0.1 g La_2O_3; background atmosphere, water vapor and 50 torr Ar; light source: xemon lamp, 300 W, $\lambda > 420$ nm. (b) *J-E* curves for Mo:BiVO_4 photoanodes with/without CoO_x in the dark (dash cuves) and under illumination (solid curves). Reaction conditions: reactant solution, 0.1 M KPi (pH = 7.2) purged with a N_2 flow; light source: Xenon lamp solar simulator, 100 mW/cm^2, AM 1.5G.

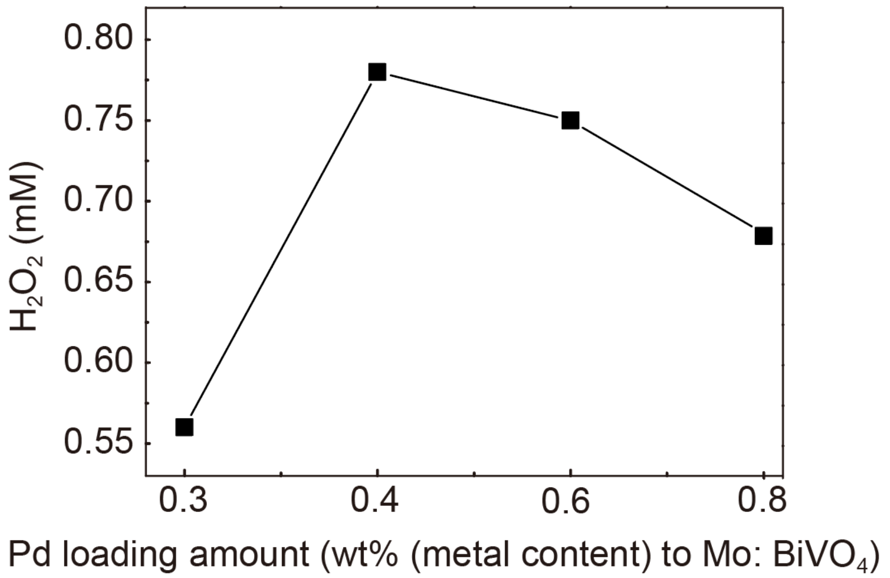

Figure S10. Amounts of H_2O_2 generation over CoO_x/Mo:$BiVO_4$/Pd as a function of the Pd loading amount. Reaction conditions: photocatalyst amount, 2 mg; reactant solution, 12 ml PBS aqueous solution (pH=7.4) saturated with O_2; light source, xenon lamp solar simulator, 100 mW/cm^2, AM 1.5G.

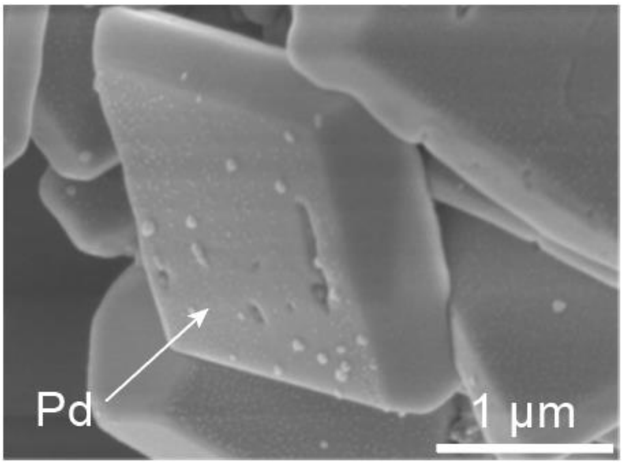

Figure S11. Scanning electron microscopy (SEM) image of Mo:$BiVO_4$/Pd. The image clearly shows that Pd nanoparticles was selectively photodeposited on the {010} facet of Mo:$BiVO_4$. The Pd loading amounts was 0.4 wt%.

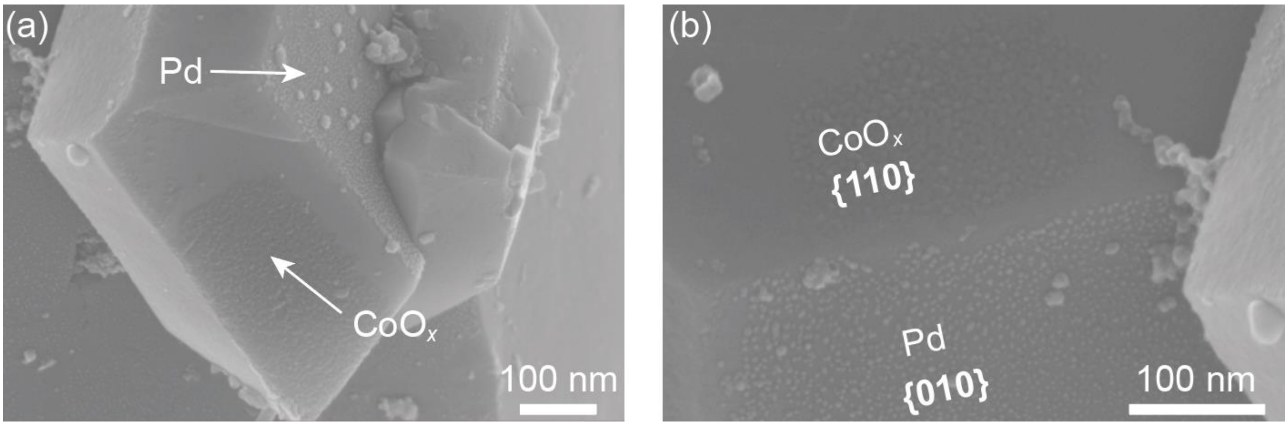

Figure S12. SEM images of CoO_x/Mo:$BiVO_4$/Pd showing selective loadings of CoO_x and Pd distribution on {110} and {010} facet, respectively.

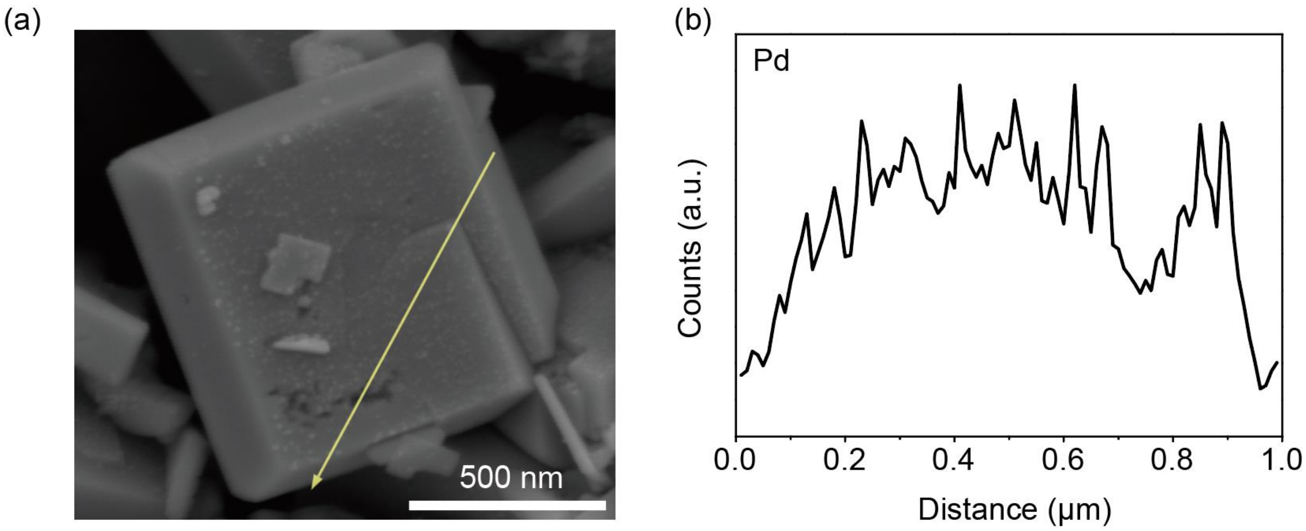

Figure S13. (a) SEM image of Mo:$BiVO_4$/Pd. (b) EDS line profile of Pd along with the yellow arrow of Mo:$BiVO_4$/Pd.

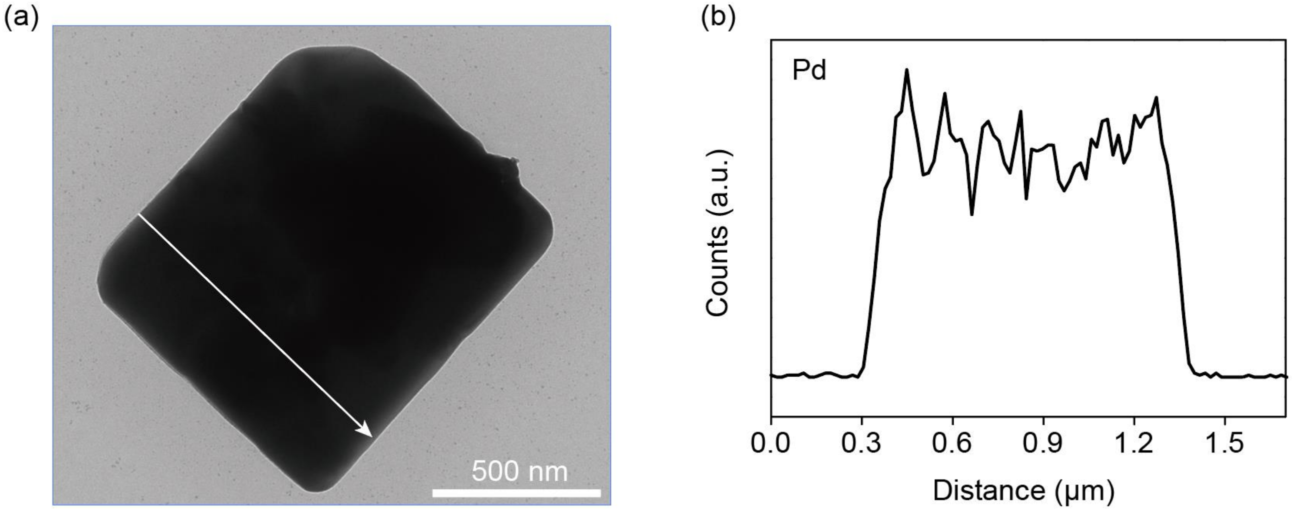

Figure S14. (a) TEM image of Mo:$BiVO_4$/Pd. (b) EDS line profile of Pd along with the white arrow of Mo:$BiVO_4$/Pd.

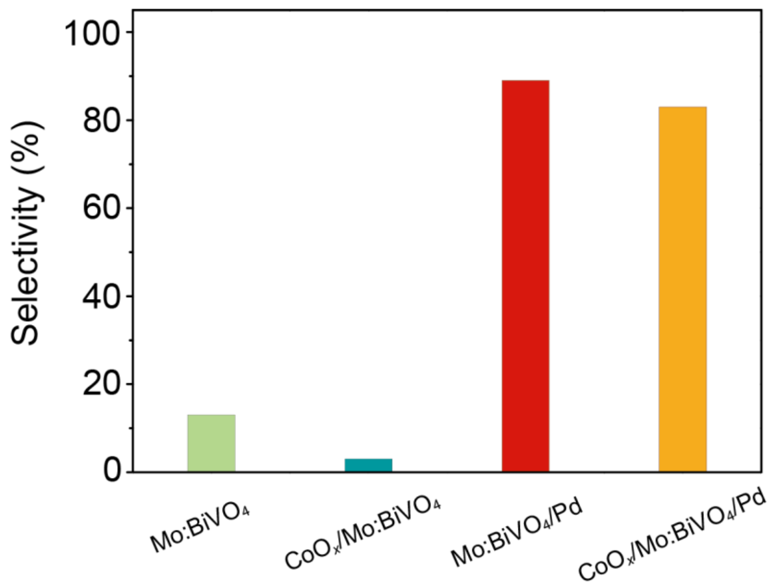

Figure S15. Selectivity of H_2O_2 production. Reaction conditions: photocatalyst amount, 2 mg; reactant solution, 12 mL PBS solution (pH=7.4) saturated with O_2; light source, xenon lamp solar simulator, 100 mW/cm^2, AM 1.5G.

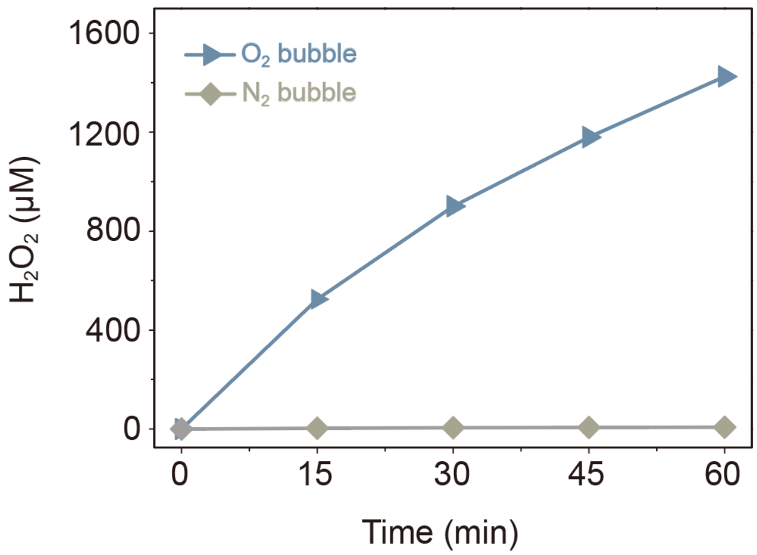

Figure S16. Amounts of H_2O_2 generation over CoO_x/Mo:$BiVO_4$/Pd under O_2- or N_2-saturated condition. The loading amounts of CoO_x and Pd were 0.2 wt% and 0.4 wt%, respectively. Reaction conditions: photocatalyst amount, 2 mg; reactant solution, 12 ml PBS aqueous solution (pH=7.4) saturated with O_2; light source, xenon lamp solar simulator, 100 mW/cm^2, AM 1.5G.

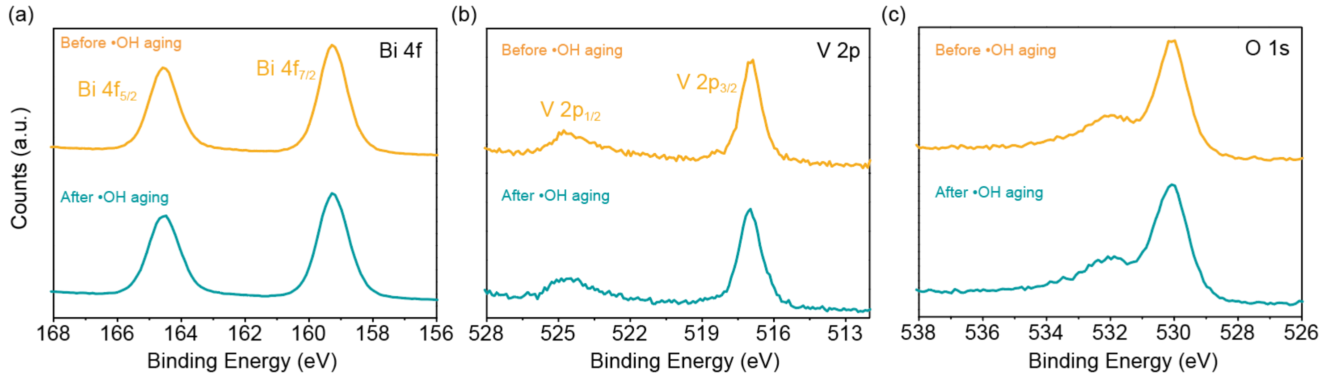

Figure S17. The Bi, V and O XPS spectra of CoO_x/Mo:$BiVO_4$/Pd before and after •OH aging for 24h.

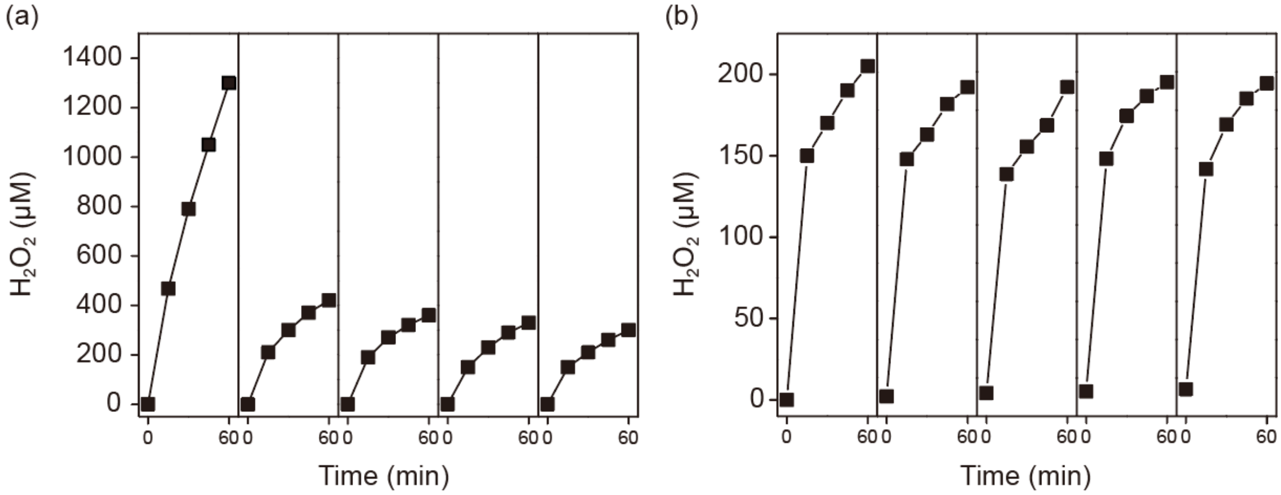

Figure S18. Time courses of photocatalytic H_2O_2 generation over CoO_x/Mo:$BiVO_4$/Pd in (a) KPi aqueous solution and (b) pure water. The loading amounts of CoO_x and Pd were 0.2 wt% and 0.4 wt%, respectively. The suspension was recycled with 0.22 μm membrance after each cycle. Reaction conditions: photocatalyst amount, 2 mg; reactant solution 12 ml, pH=7.4, solution saturated with O_2; light source: xenon lamp solar simulator, 100 mW/cm^2, AM 1.5G.

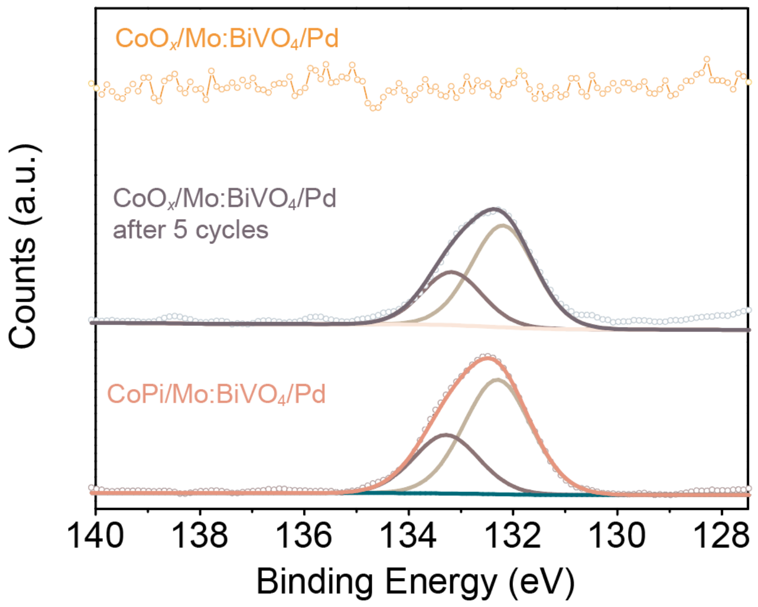

Figure S19. P 2p XPS spectra of CoO_x/Mo:$BiVO_4$/Pd (yellow), CoO_x/Mo:$BiVO_4$/Pd (brown) after 5 cycles of photocatalytic H_2O_2 generation in 1M PO_4^{3-} solution and CoPi/Mo:$BiVO_4$/Pd (orange).

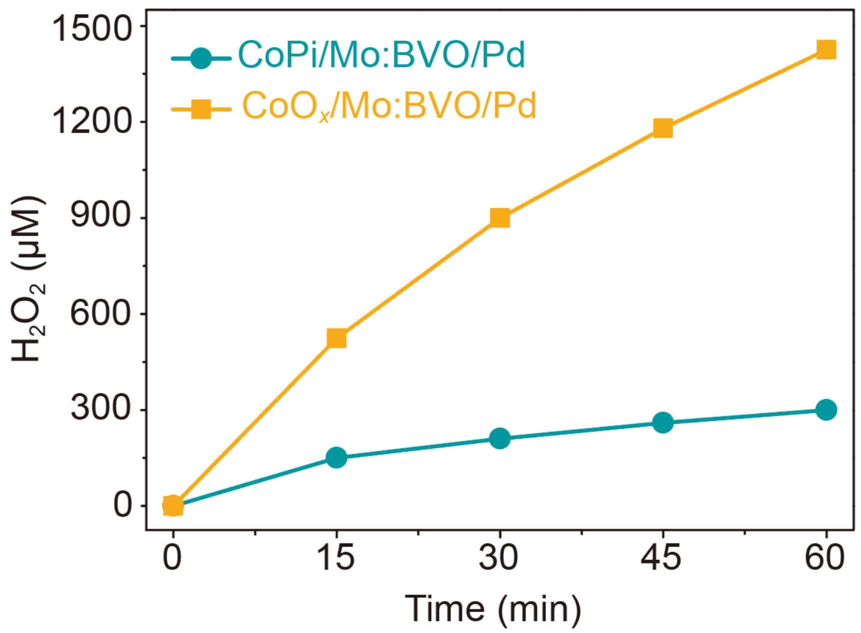

Figure S20. Photocatalytic H_2O_2 production by CoPi/Mo:$BiVO_4$/Pd and CoO_x/Mo:$BiVO_4$/Pd. Reaction conditions: photocatalyst amount, 2 mg; reactant solution, 12 ml PBS aqueous solution, pH=7.4 saturated with O_2; light source, xenon lamp solar simulator, 100 mW/cm^2, AM 1.5G. The H_2O_2 generation in one hour for CoO_x/Mo:$BiVO_4$/Pd and CoPi/Mo:$BiVO_4$/Pd were 1425 μM and 299 μM, respectively. CoP_i/Mo:$BiVO_4$ was prepared by dispersing 0.2 g $BiVO_4$ powder in 100 ml PO_4^{3-} (1 M) and $NaIO_3$ solutions (0.01 M, pH=7.4), followed by addition of 0.28 ml $Co(NO_3)_2$ solution (1.5 g/L). The mixture solution was irradiated with a 300 W Xe lamp (≥420 nm) under continuous stirring for 3 hours, filtered, washed with deionized water for 3 times, and dried at 60 °C for 8 h. CoP_i/Mo:$BiVO_4$/Pd was prepared by dispersing 0.2 g CoPi/Mo:$BiVO_4$ in 100 ml pure water, followed by addition of 0.24 ml Na_2PdCl_4 solution (3.3 g/L). The mixture solution was irradiated with a 300 W Xe lamp (≥420 nm) under continuous stirring for 3 h, filtered, washed with deionized water for 3 times, and dried at 60 °C for 8 h.

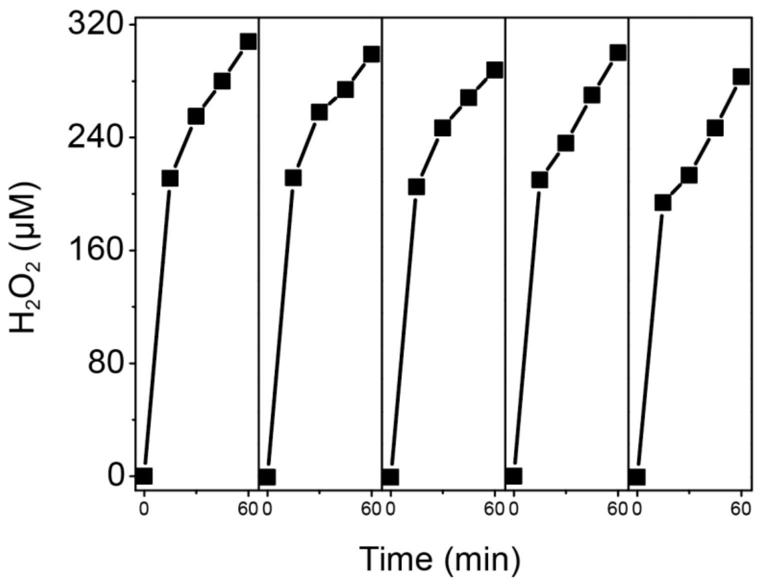

Figure S21. Time courses of photocatalytic H_2O_2 generation over CoO_x/Mo:$BiVO_4$/Pd in sea water. The seawater is prepared by dissolving the sea salt consisted with NaCl (77.6 wt%), KCl (2.1 wt%), $MgCl_2$ (6.6 wt%), $CaCl_2$ (3.3 wt%), $MgSO_4$ (9.6 wt%), $NaHCO_3$ (0.6 wt%), NaBr (0.16 wt%), Na_2SiO_3 (0.007 wt%), $NaSi_4O_9$ (0.006 wt%), H_3PO_4 (0.006 wt%), H_3BO_3 (0.2 wt%), $LiNO_3$ (0.004 wt%), Al_2Cl_6 (0.04 wt%) in the DI water.

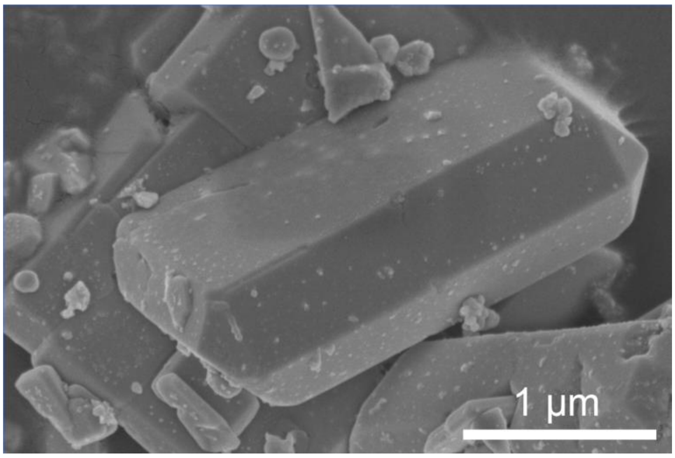

Figure S22. SEM image of Mo:$BiVO_4$-CoO_x-Pd. The loading amounts of CoO_x and Pd were 0.2 wt% and 0.4 wt%, respectively. The image shows that uncontrolled deposition of CoO_x and Pd on Mo:$BiVO_4$ by impregnation method resulted in random deposition of CoO_x and Pd on Mo:$BiVO_4$ without facet selectivity.

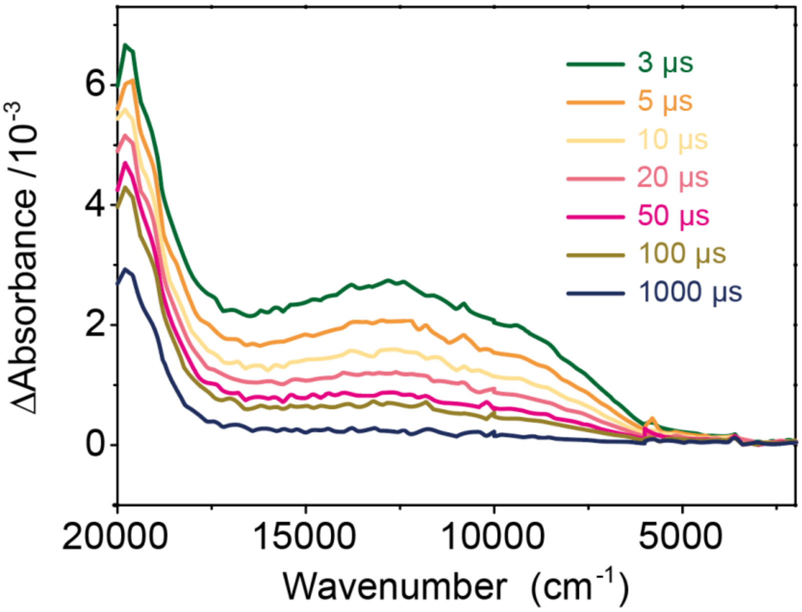

Figure S23. TA spectra of Mo:$BiVO_4$ irradiated with laser pulses (duration: 6 ns, fluence: 3 mJ pulse^{-1}) in vacuum. The strong absorption at 20000 – 17000 cm^{-1} is attributed to trapped holes as reported previously on Mo:$BiVO_4$ photocatalysts.[12-14] The broad absorption from 170000 – 5000 cm^{-1} is assigned to deeply trapped electrons since the TA intensity at this region decreased when Pd as an electron sink was loaded on Mo:$BiVO_4$ (Figure S24). The absorption < 5000 cm^{-1} was reported to be free/shallowly trapped electrons.[15]

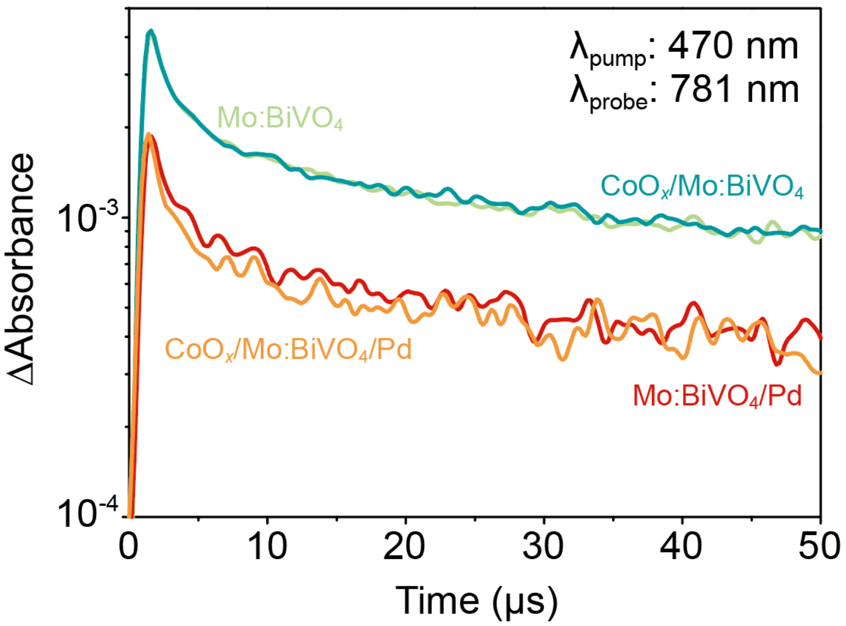

Figure S24. Transient profiles of photocarriers probed at 781 nm (deeply trapped electrons) for $Mo:BiVO_4$, $Mo:BiVO_4/Pd$, $CoO_x/Mo:BiVO_4$ and $CoO_x/Mo:BiVO_4/Pd$.

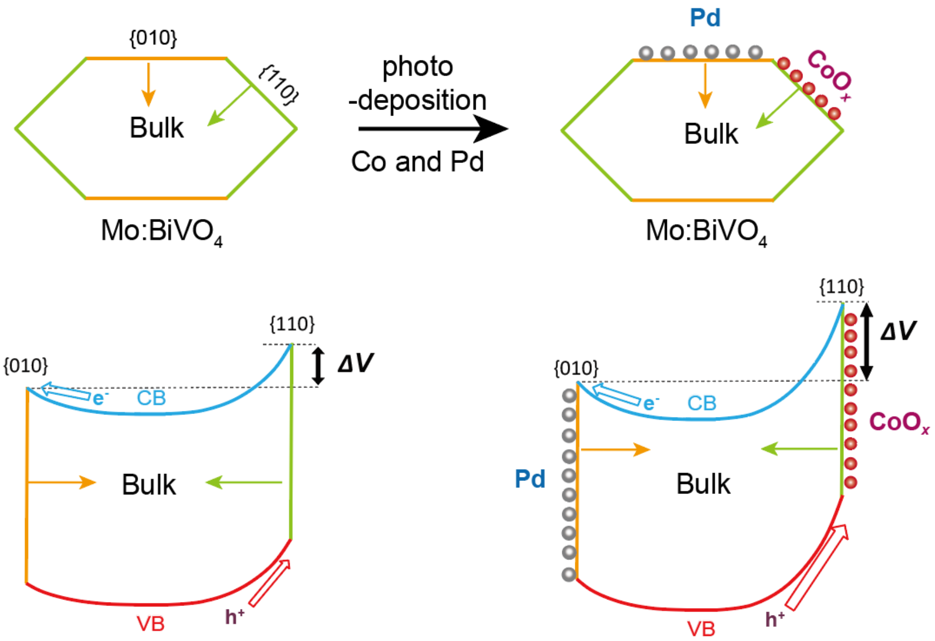

Figure S25. Schematic band diagrams across the border between the {011} and {010} facets of a bare single $Mo:BiVO_4$ photocatalyst particle and facets of a single $Mo:BiVO_4$ photocatalyst particle with CoO_x cocatalyst selectively deposited on {010} facet (green line) and Pd cocatalyst selectively deposited on {010} facet (orange line), respectively. The facet dependent energetics on a bare single $Mo:BiVO_4$ photocatalyst particle was caused by the variation in surface termination atoms.[16]

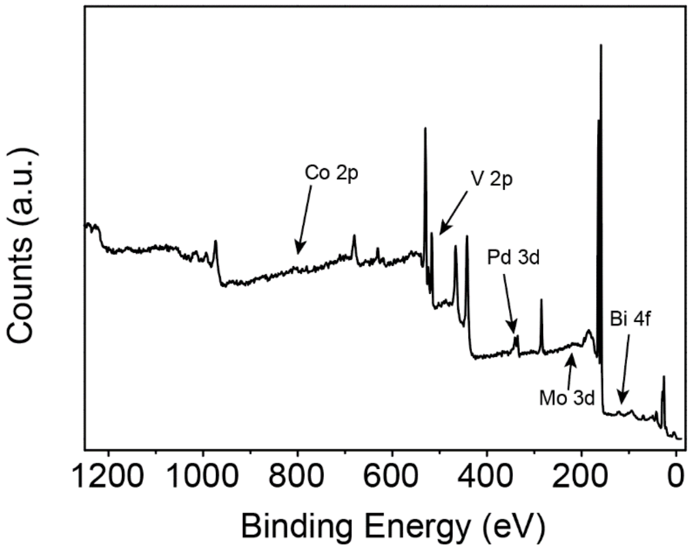

Figure S26. The full XPS spectrum of CoO_x/Mo:$BiVO_4$/Pd.

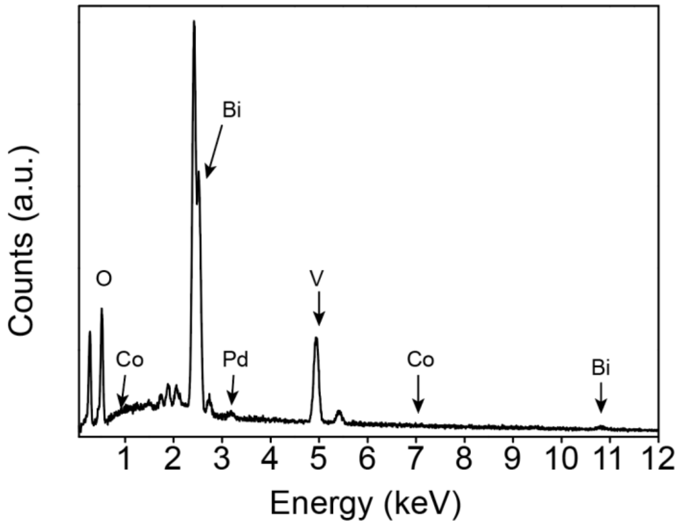

Figure S27. The EDX spectrum of CoO_x/Mo:$BiVO_4$/Pd.

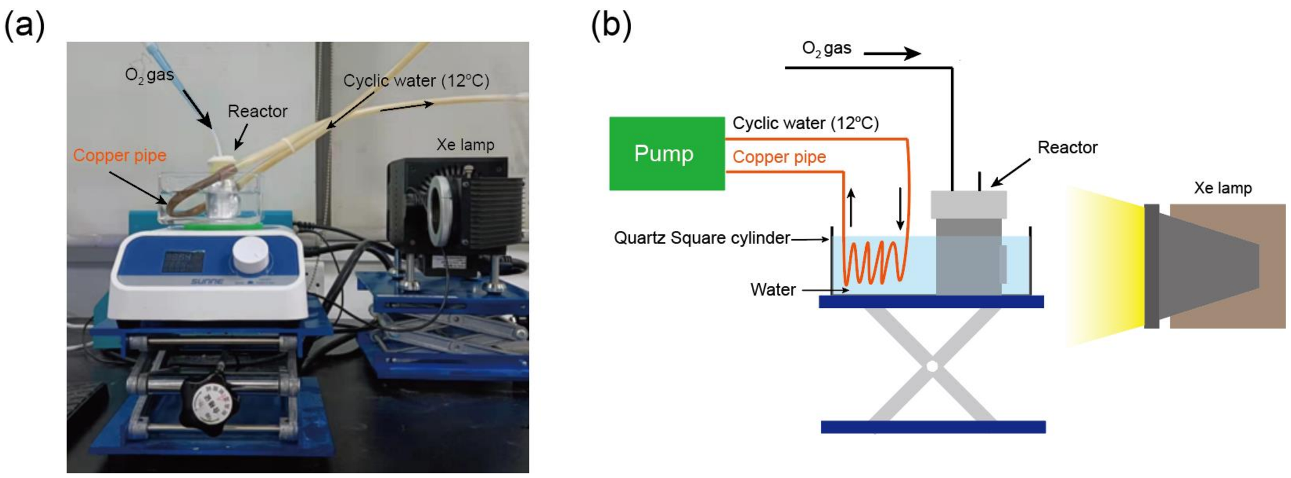

Figure S28. (a) Photographs and (b) schematic illustration of the devices used in the photocatalysis performance tests.

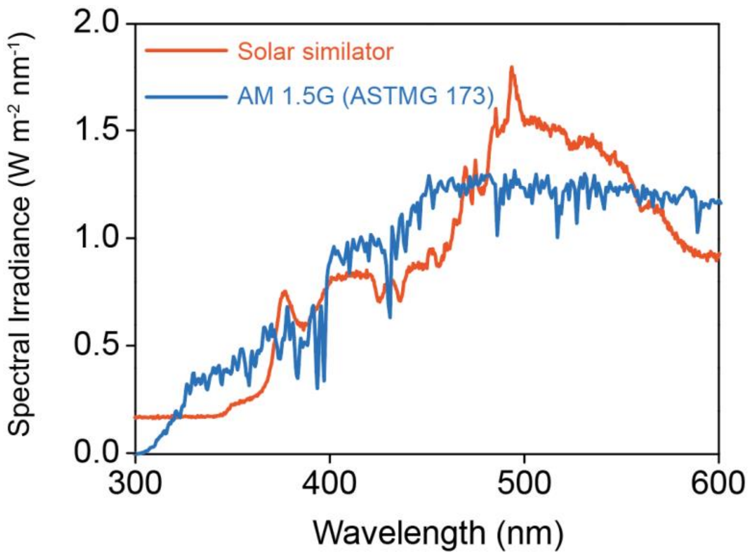

Figure S29. Spectrum of the xenon lamp solar simulator (orange line) and the standard AM 1.5G (blue line, ASTMG 173).

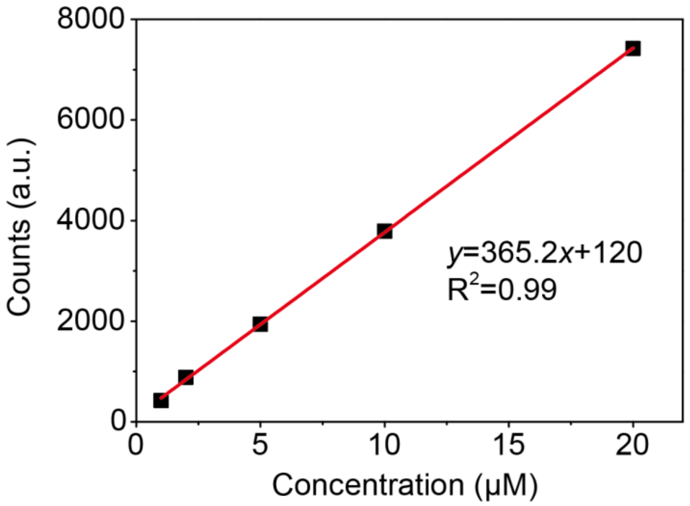

Figure S30. Calibration curve for quantifying photogenerated H_2O_2. The H_2O_2 concentration was calculated following the equation: Counts = 365.2[H_2O_2] + 120. For example, HPLC analysis of 60-min H_2O_2 production by CoO_x/Mo:$BiVO_4$/Pd gave a signal of 6620, corresponding to a H_2O_2 concentration of 17.8 μM. Because we diluted the H_2O_2 solution by 80 times before analysis, the photogenerated H_2O_2 was 1425 μM.

Supplementary References

1 Chu, C. H. *et al.* Spatially separating redox centers on 2D carbon nitride with cobalt single atom for photocatalytic H_2O_2 production. *Proc. Natl. Acad. Sci. U.S.A.* **117**, 6376-6382 (2020).

2 Chu, C. H. *et al.* Electronic tuning of metal nanoparticles for highly efficient photocatalytic hydrogen peroxide production. *Acs Catal.* **9**, 626-631 (2019).

3 Moon, G. H., Kim, W., Bokare, A. D., Sung, N. E. & Choi, W. Solar production of H_2O_2 on reduced graphene oxide-TiO_2 hybrid photocatalysts consisting of earth-abundant elements only. *Energy Environ. Sci.* **7**, 4023-4028 (2014).

4 Hou, W. C. & Wang, Y. S. Photocatalytic generation of H_2O_2 by graphene oxide in organic electron donor-free condition under sunlight. *Acs Sustain. Chem. Eng.* **5**, 2994-3001 (2017).

5 Hirakawa, H. *et al.* Au nanoparticles supported on $BiVO_4$: effective inorganic photocatalysts for H_2O_2 production from water and O_2 under visible light. *Acs Catal.* **6**, 4976-4982 (2016).

6 Shiraishi, Y. *et al.* Resorcinol-formaldehyde resins as metal-free semiconductor photocatalysts for solar-to-hydrogen peroxide energy conversion. *Nat. Mater.* **18**, 985-993 (2019).

7 Teng, Z. *et al.* Atomically dispersed antimony on carbon nitride for the artificial photosynthesis of hydrogen peroxide. *Nat. Catal.* **4**, 374-384, (2021).

8 Kofuji, Y. *et al.* Graphitic carbon nitride doped with biphenyl diimide: efficient photocatalyst for hydrogen peroxide production from water and molecular oxygen by sunlight. *Acs Catal.* **6**, 7021-7029 (2016).

9 Kofuji, Y. *et al.* Carbon nitride-aromatic diimide-graphene nanohybrids: metal-free photocatalysts for solar-to-hydrogen peroxide energy conversion with 0.2% efficiency. *J. Am. Chem. Soc.* **138**, 10019-10025 (2016).

10 Zhu, Z. D., Pan, H. H., Murugananthan, M., Gong, J. Y. & Zhang, Y. R. Visible light-driven photocatalytically active g-C_3N_4 material for enhanced generation of H_2O_2. *Appl. Catal. B Environ.* **232**, 19-25 (2018).

11 Ye, Y. X. *et al.* Highly efficient photosynthesis of hydrogen peroxide in ambient conditions. *Proc.. Natl. Acad. Sci. USA.* **118** (2021).

12 Ma, Y. M., Pendlebury, S. R., Reynal, A., Le Formal, F. & Durrant, J. R. Dynamics of photogenerated holes in undoped $BiVO_4$ photoanodes for solar water oxidation. *Chem. Sci.* **5**, 2964-2973 (2014).

13 Yamakata, A., Ranasinghe, C. S. K., Hayashi, N., Kato, K. & Vequizo, J. J. M. Identification of individual electron- and hole-transfer kinetics at $CoO_x/BiVO_4/SnO_2$ double heterojunctions. *Acs Appl. Energ. Mater.* **3**, 1207-1214 (2020).

14 Suzuki, Y. *et al.* Rational interpretation of correlated kinetics of mobile and trapped charge carriers: analysis of ultrafast carrier dynamics in $BiVO_4$. *J. Phys. Chem. C* **121**, 19044-19052 (2017).

15 Vequizo, J. J. M. *et al.* Trapping-induced enhancement of photocatalytic activity on brookite TiO_2 powders: comparison with anatase and rutile TiO_2 powders. *Acs Catal.* **7**, 2644-2651 (2017).

16 Yang, H. G. *et al.* Anatase TiO_2 single crystals with a large percentage of reactive facets. *Nature* **453**, 638-U634 (2008).
